# Magnetic Microrobots for In Vivo Cargo Delivery: A Review

**DOI:** 10.3390/mi15050664

**Published:** 2024-05-20

**Authors:** Jialin Lin, Qingzheng Cong, Dandan Zhang

**Affiliations:** Department of Bioengineering, Imperial College London, Exhibition Road, South Kensington, London SW7 2AZ, UK; j.lin@imperial.ac.uk (J.L.); q.cong@imperial.ac.uk (Q.C.)

**Keywords:** magnetic microrobot, in vivo cargo delivery, biomedicine

## Abstract

Magnetic microrobots, with their small size and agile maneuverability, are well-suited for navigating the intricate and confined spaces within the human body. In vivo cargo delivery within the context of microrobotics involves the use of microrobots to transport and administer drugs and cells directly to the targeted regions within a living organism. The principal aim is to enhance the precision, efficiency, and safety of therapeutic interventions. Despite their potential, there is a shortage of comprehensive reviews on the use of magnetic microrobots for in vivo cargo delivery from both research and engineering perspectives, particularly those published after 2019. This review addresses this gap by disentangling recent advancements in magnetic microrobots for in vivo cargo delivery. It summarizes their actuation platforms, structural designs, cargo loading and release methods, tracking methods, navigation algorithms, and degradation and retrieval methods. Finally, it highlights potential research directions. This review aims to provide a comprehensive summary of the current landscape of magnetic microrobot technologies for in vivo cargo delivery. It highlights their present implementation methods, capabilities, and prospective research directions. The review also examines significant innovations and inherent challenges in biomedical applications.

## 1. Introduction

Targeted cargo delivery, encompassing the precise transportation of drugs and cells using microrobots within biological systems, is at the forefront of modern medical innovation. Unlike traditional delivery methods that often distribute drugs systemically, microrobots can deliver therapeutic agents directly to specific tissues. This capability is particularly beneficial for accessing hard-to-reach areas within the body, thereby enhancing the overall effectiveness of the treatment while minimizing the potential side effects. Also, by enabling targeted therapies through less invasive methods, they reduce the physical pain in patients, leading to faster recovery times and fewer complications post-treatment [1,2].

Although some studies define microrobots as ranging in size from micrometers to nanometers [3]. It is important to emphasize that microrobots within the micrometer range are particularly suitable for in vivo targeted cargo delivery. This preference is due to their larger capacity for carrying cargo compared to nanometer-sized robots [4,5]. Additionally, the micrometer-sized robots allow for easier tracking using conventional imaging technologies, resulting in lower costs since high-resolution detection is unnecessary [6]. Moreover, micrometer-sized robots are less likely to accumulate in the human body, thereby reducing potential toxicity [6,7]. Consequently, in this review, we define ‘microrobot’ as robots with micrometer dimensions, rather than those on the nanoscale.

Microrobots can be actuated by magnetic, acoustic, and optical fields [3,8,9,10]. Magnetic fields have emerged as a critical element in the control and operation of microrobots, especially in the realm of in vivo medical applications. They are capable of precisely controlling the movements and functions of microrobots [11,12,13]; moreover, low-strength magnetic fields can safely penetrate biological tissues without significant attenuation [14]. These unique properties make magnetic microrobots (MMRs) ideal for in vivo targeted therapeutic interventions.

To actuate MMRs, a variety of magnetic field generators can be employed, including permanent magnets [15,16,17], electromagnets [18], and Helmholtz and/or Maxwell coils [19,20,21,22]. These devices can produce a uniform or non-uniform, gradient [23], or rotating magnetic fields [24,25], exerting forces or torques [26] (or a combination thereof) on ferromagnetic [27] or paramagnetic microrobots [28]. While the actuation platforms may differ, the fundamental physical principles remain consistent. To facilitate the understanding of MMR actuation methods, this paper reviews the theoretical foundation, followed by several common platforms used in recent advancements of MMRs for in vivo cargo delivery.

The high viscosity of blood and the micrometer size of MMRs lead to an environment where viscous forces dominate over inertial forces [29]. This complicates the structural design of MMRs since geometrically reciprocal motion cannot produce displacement in such conditions [30]. Therefore, the careful structural design of MMRs is necessary to break geometric symmetry and further achieve net displacement for effective cargo delivery. Additionally, specific designs must address their particular fabrication and functional needs.

MMRs also stand out for their controlled release capabilities, since they can release drugs in response to specific physiological triggers or external stimuli. This responsiveness ensures that the therapeutic agents exert their effects only where and when needed, which is crucial for maximizing efficacy and minimizing adverse reactions. They can dynamically adjust treatment protocols based on immediate physiological responses. This adaptability enhances the precision of medical interventions, making treatments more effective and tailored to individual patient needs. Furthermore, their navigational abilities enhanced by Artificial Intelligence (AI) could allow them to maneuver precisely through intricate in vivo environments, such as narrow blood vessels.

Despite the promising advances in the field of MMRs for in vivo cargo delivery, several challenges persist, which hinder their widespread clinical adoption. These challenges include achieving effective actuation, determining the proper structure, loading and releasing therapeutic cargo, tracking and navigating MMRs in complex body environments, and ensuring their biocompatibility.

The effectiveness of external magnetic fields in controlling MMRs diminishes with depth and tissue interference, complicating their deployment in deep-tissue or highly sensitive areas. This necessitates the careful design of magnetic actuation platforms to meet the needs of specific deployment scenarios. This challenge also extends to the MMR’s structural design and cargo loading and release methods. For various MMRs fabrication requirements, cargo types, and target delivery sites, MMRs’ design and deployment are still in the time-consuming trial-and-error stage. The effective real-time monitoring and control of MMRs within the body are crucial for successful cargo delivery. This necessitates the use of advanced imaging and tracking systems capable of operating at the necessary scale and resolution for the navigation of MMRs [31]. Navigating an MMR to a precise location within the human body’s complex and dynamic environment presents considerable technical challenges. These challenges include overcoming the hurdles posed by body fluids, varying tissue densities, and intricate anatomical structures [32]. Biocompatibility is a critical concern for the use of MMRs in medical applications. The materials used in these devices must be non-toxic and biocompatible to prevent adverse immune reactions. It is crucial to thoroughly understand and optimize the long-term interactions between these materials and biological tissues. Additionally, after treatment, it is essential to ensure the safe removal or biodegradation of MMRs. The accumulation of non-biodegradable materials in the body could lead to serious complications, making it necessary to design MMRs that can either degrade harmlessly or be effectively retrieved [33]. This ensures that the MMRs perform their intended function without posing long-term health risks.

**Figure 1:**
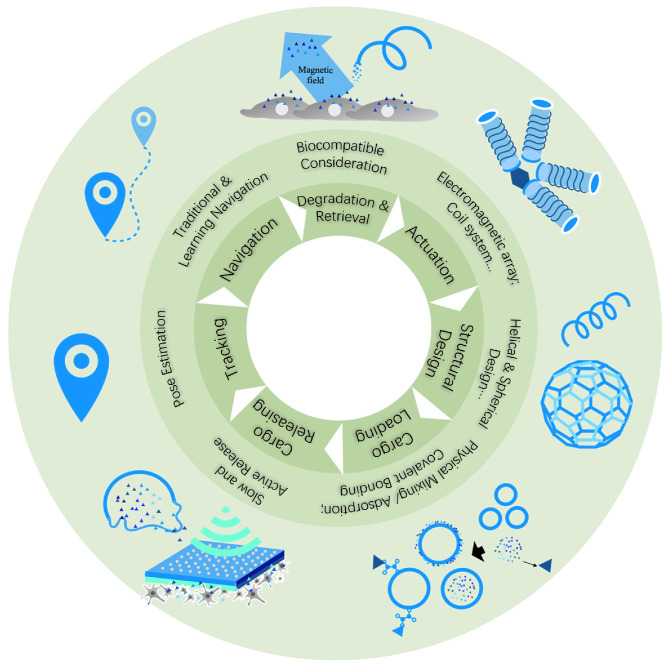
The concept figure of this review. We systematically decouple recent works on MMRs for in vivo cargo delivery into a structured framework. The framework includes magnetic field actuation, structural design, cargo loading and release mechanisms, tracking, navigation, as well as the degradation and retrieval of MMRs.

In response to these challenges, many studies have been conducted. Notably, there is a noticeable shortfall in comprehensive reviews on MMRs for in vivo cargo delivery, especially those published after 2019. As depicted in the concept figure (Figure 1), this review aims to examine the recent advancements in MMRs for in vivo cargo delivery, viewed through an engineering and application lens. We adopt a structured approach in our review, introducing the magnetic field actuation, structural design, cargo loading and release mechanisms, tracking, navigation, and the degradation and retrieval of MMRs. Our goal is to disentangle these elements from the recent works of the past five years, thereby offering a clear, streamlined review. Additionally, we identify the current challenges and future opportunities in the field. We hope this structured overview will empower new researchers to contribute significantly to and enhance the community.

## 2. Magnetic Actuation Method

In the context of MMRs for in vivo cargo delivery, the first consideration should be how to properly actuate them by controlling the magnetic field. Therefore, we first delve into the actuation theory, and then review typical magnetic actuation platforms for MMRs.

### 2.1. Actuation Principle

Since James Clerk Maxwell’s seminal amendment to Ampère’s law, introducing a displacement current to account for dynamic electromagnetic fields, Maxwell’s equations have served as the cornerstone of electrodynamics for nearly two centuries. These principles have been rigorously validated, proving their applicability even within the micro-scale, high-viscosity fluid environments of the human body. This solid theoretical foundation underpins the use of magnetic fields for propelling MMRs designed for in vivo cargo delivery tasks.

Theoretically, MMR is usually treated as a magnetized object in the magnetic field, defined by a magnetic moment **M** comprising three components: $m_{x}$, $m_{y}$, and $m_{z}$, each measured in ampere-meter squared ($A\cdot m^{2}$). This magnetic moment, resulting from a permanent magnet embedded within the MMR, is expected to have a constant magnitude and to be securely affixed to the MMR’s structure. For the sake of simplifying engineering calculations, it is common practice to transform the vector cross product into matrix multiplication. This is achieved by converting **B** into a skew-symmetric matrix, denoted as Sk(**B**). Numerous studies have provided equations for calculating the torque and force in such systems, and our calculations adhere to these established methods [18,28,34,35]. Therefore, we can accurately compute the torque experienced by the MMR in a magnetic field:

(1) $$T=M\times B=\mathrm{Sk}\left(B\right)M=\left[\begin{array}{ccc} 0 & -B_{z} & B_{y}\\ B_{z} & 0 & -B_{x}\\ -B_{y} & B_{x} & 0 \end{array}\right]\left[\begin{array}{c} m_{x}\\ m_{y}\\ m_{z} \end{array}\right]$$

where $B\in R^{3}$ is induced magnetic field strength. Considering various magnetic field intensities (H) and the relative permeability to vacuum ($\mu_{r}$), the induced magnetic field strength can be calculated as $B=\mu_{r}H$.

The force experienced by the microrobot within a magnetic field can be expressed as follows:

(2) $$F=\left(M\cdot \nabla \right)B=\left[\begin{array}{ccc} \frac{\partial B_{x}}{\partial x} & \frac{\partial B_{x}}{\partial y} & \frac{\partial B_{x}}{\partial z}\\ \frac{\partial B_{y}}{\partial x} & \frac{\partial B_{y}}{\partial y} & \frac{\partial B_{y}}{\partial z}\\ \frac{\partial B_{z}}{\partial x} & \frac{\partial B_{z}}{\partial y} & \frac{\partial B_{z}}{\partial z} \end{array}\right]\left[\begin{array}{c} m_{x}\\ m_{y}\\ m_{z} \end{array}\right]$$

where *∇* represents the Del operator, this equation signifies that the magnetic force B is determined by the rate of change in the magnetic field B along the direction of the magnetic moment M.

Based on these two fundamental equations, a mapping relationship between the magnetic field and the actuation force and torque can be established. The fundamental equations refer to the principles governing magnetic fields and forces in both permanent magnets and electromagnets. For permanent magnets, the magnetic field can be determined from their material properties, shapes, and dimensions. In the case of electromagnets or coil systems, the Biot–Savart law, which describes the magnetic field generated by an electric current, can be used to establish the relationship between current and the strength of magnetic fields [36]. Therefore, by adjusting the current in the electromagnet, the magnitude or direction of the magnetic force and torque can be controlled. This ability to control magnetic forces precisely is crucial for applications requiring targeted and adjustable actuation.

### 2.2. Actuation Platform

To generate magnetic fields for MMR propulsion, many platforms have been established. These platforms can generally be categorized into three types: electromagnet arrays, Maxwell/Helmholtz coil systems, and permanent magnet setups.

**Figure 2:**
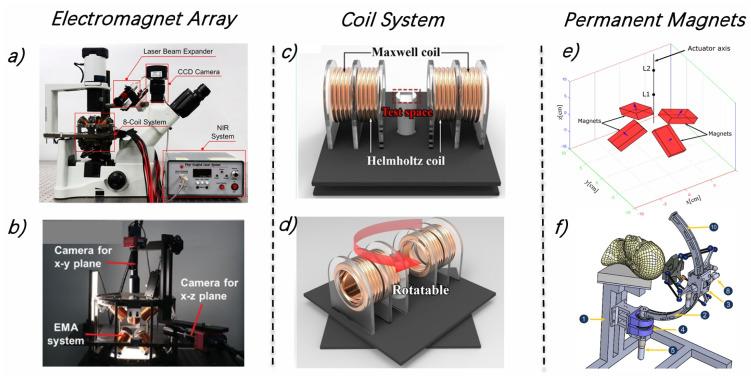
Typical magnetic actuation platforms for MMRs. (**a**,**b**) Electromagnet array platform offers 5 degrees of freedom (5-DoF) for precise control of MMRs, enabling complex manipulation. (**c**,**d**) The coil system for MMR actuation features a rotatable mechanism, simplifying the 2D coil actuation system. Without the rotatable mechanism, an additional set of Maxwell and Helmholtz coils is needed. (**e**,**f**) Permanent magnet array actuation system: since permanent magnets cannot vary the magnitude of their magnetic fields, these systems typically require an additional robot to move the permanent magnet array. This movement generates a magnetic field gradient, which is crucial for driving MMRs. Figures adapted with permissions from ref. [48], ACS, (**a**); ref. [49], Wiley, (**b**); ref. [41], Elsevier, (**c**,**d**); ref. [17], MDPI, (**e**,**f**).

Among those, OctoMag and MiniMag [18,37], representing electromagnet array configurations, are the most extensively utilized across all platforms [38,39,40] (see examples in Figure 2a,b). This design offers enhanced precision and flexibility in control by adjusting the intensity and direction of the current through the electromagnets, enabling the generation of complex magnetic fields for 5-DOF micromanipulation of MMRs.

Coil systems, including those based on Maxwell and Helmholtz designs [41,42,43,44,45] (see examples in Figure 2c,d), offer a simpler design compared to electromagnets and can provide more uniform magnetic fields. However, these designs tend to occupy more space, resulting in smaller operational workspaces, which may not be practical for actual in vivo treatments. For instance, the coil system proposed by Jeong et al. [46], despite its simple structure, ease of design of magnetic actuation platform, has a limited 2D workspace of only 20.6 × 20.6 mm. For in vivo applications, the volume of the coils would significantly increase.

Although these electromagnetic actuator systems can generate magnetic fields on demand, they require high power consumption, leading to substantial heat release in the workspace and necessitating cooling systems [16]. Replacing electromagnetic devices with permanent magnets can produce bigger magnetic fields magnitude and gradients, enhanced by 10–20 times and 2–3 times, respectively [47].

Permanent magnets with special configurations can be used to create magnetic field gradients as propulsion platforms in a flexible way. As shown in Figure 2e,f, Abbes et al. proposed a magnetic propulsion system composed of four permanent magnets to drive MMRs [16,17]. Compared to configurations with dual permanent magnets [50], this setup exhibits symmetrical behavior in both the xz and yz planes, allowing for the generation of magnetic forces converging to fixed points on both planes, which is beneficial to MMRs’ control. Compared to electromagnetic systems, permanent magnet systems offer greater flexibility and larger workspaces. However, using permanent magnets to propel MMRs requires additional robots to move the magnets, adding complexity to the system’s design.

## 3. Structural Design

For cargo delivery tasks, such as drug or cell delivery, the structural design of MMRs presents substantial challenges because designers must consider the MMRs’ ability to navigate fluidic environments while maintaining sufficient cargo carrying capacity.

The Reynolds number (Re), defined as $Re=\frac{UL\rho }{\eta }$, where *U* and *L* represent velocity and characteristic length, respectively, and ρ and η denote the fluid’s density and viscosity, measures the ratio of inertial forces to viscous forces within a fluid. At low Re numbers (Re < 1), the inertial forces are negligible and viscous forces dominate. Given the small values of *U* and *L* at the micro-scale, Re numbers are significantly less than one, even in fluids not typically considered viscous, such as water. The scallop theorem indicates that at low Re numbers, geometry rather than speed is crucial and reciprocal motion does not result in net displacement. [51]. To disrupt the non-reciprocal motion of MMRs and generate net displacement for in vivo cargo delivery, a common strategy is to design MMRs with asymmetric structures. Additionally, when MMRs need to carry cells or other specific functions, spherical and deformable designs are usually adopted.

### 3.1. Helical Design

**Figure 3:**
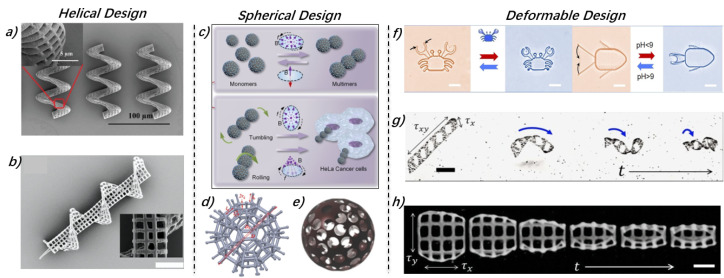
Typical structural design of MMRs. (**a**) The porous helical design of MMRs significantly enhances their magnetism, and thus their controllability in viscous fluids by increasing the surface area. This feature greatly improves cargo loading and release capabilities and magnetic interaction efficiency. (**b**) Needle and helical design, it can fix to the target organ or tissue. (**c**) Spherical MMRs possess the unique ability to reconfigure in response to specific magnetic fields, showcasing versatile operational modes. (**d**,**e**) Spherical scaffolds MMRs for cell transportation. (**f**) MMRs made from PH-sensitive gel, enabling them to deform and adapt to different environmental conditions effectively. (**g**,**h**) The design of self-folding MMRs incorporates a folding mechanism that significantly accelerates their fabrication process. Figures adapted with permissions from ref. [60], Wiley, (**a**); ref. [40], Wiley, (**b**); ref. [42], ACS, (**c**); ref. [63], Wiley, (**d**); ref. [64], Wiley, (**e**); ref. [65], ACS, (**f**); ref. [66], Elsevier, (**g**); ref. [67] Wiley, (**h**).

Helical MMRs, in particular, are widely utilized for the delivery of therapeutic drugs in highly viscous fluids such as blood, demonstrating the effective movement in environments with low Reynolds numbers [52,53]. Helical MMRs had been well developed theoretically. Wang et al. conducted dynamic modeling of magnetically driven helical MMRs, analyzing their swimming characteristics and the impact of different designs on their swimming speed, step-out frequency, and maximum velocity [54]. Samsami et al. derived and validated an analytical criterion to test the stability of steady solutions for rigid helical MMRs made of soft magnetic materials, actuated by a rotating magnetic field [55]. Drawing on insights from previous studies on actuating helical MMRs in viscous fluids, recent research increasingly adopts them for cargo delivery applications. [48,56,57,58,59]. For example, Park et al. introduced porous helical MMRs (Figure 3a). The porous structure not only increases the surface area, allowing for more magnetic coating on MMRs and further leading to higher magnetism but also benefits focused ultrasound beam drug release [60]. Considering the difficulties in maintaining MMRs in a fixed position for prolonged drug delivery without a constant magnetic field, needle-type 3D MMRs featuring a combination of porous and helical structures (Figure 3b) were developed to address these challenges and enhance magnetic propulsion [40].

The unique advantage of helical design also lies in its capability to achieve independent control of multiple robots within the same area. Katsamba et al. proposed a concept for a dual-helix structured microrobot that features magnetic helices of opposite chirality. Adjusting the competitive relationship between these helices enables intrinsic non-linearity, allowing each device to operate within a specific frequency range [61]. Based on this, Giltinan et al. conducted experimental validations, demonstrating significant movement only beyond a critical frequency and a micro motor that changes its translational direction with the frequency of the rotating magnetic field. This independent control is crucial for the precise manipulation of microrobots in liquid media [62].

### 3.2. Spherical Design

The spherical MMRs design represents another commonly used structure. Similar to helical design, mathematical modeling for the propulsion of spherical microrobots in viscous fluids has been established and validated on magnetic actuation platforms. The experimental results confirmed the consistency of the proposed dynamic model, which describes the behavior of spherical MMRs under magnetic propulsion [68].

Solid spherical MMRs have been explored in earlier research [69]. Researchers have used micro magnetic particles directly as MMRs, avoiding the need for structural design. As shown in Figure 3c, MMR swarms, these magnetic spheres could be used as research subjects, simplifying individual robot design and focusing on flexible, programmable, and reconfigurable MMR swarms through magnetic field control [42,70,71]. However, these microrobots are limited to surface drug loading through chemical bonding or physical adsorption, restricting their capacity to transport cell-type cargoes.

For cell delivery, spherical scaffolds have been considered. The design of spherical scaffold microrobots can be traced back to [72,73]. As tissue culture scaffolds, their primary purpose is to achieve 3D cell incubation within the sphere, a capability not inherent to helically shaped MMRs. Li et al. developed an MMR for carrying and delivering targeted cells, using a burr-like porous spherical structure to enhance the magnetic driving and cell-carrying capabilities. Experiments showed that porous spherical structures have stronger magnetic driving capabilities compared to porous cubic structures, and the burr-like design enhances cell-carrying capacity [63,74] (Figure 3d). Go et al. developed a magnetically driven micro-scaffold designed to transport mesenchymal stem cells (MSCs) for use in joint cartilage regeneration applications [64,75]. As shown in Figure 3e, the micro-scaffold, a 3D porous microbead, supported MSC adhesion and migration, could be guided to target sites by external magnetic fields. The main body and surface of the micro-scaffold were composed of Poly(Lactic-co-Glycolic acid) (PLGA) and superparamagnetic nanoparticles (MNPs), respectively, combining biodegradability and the magnetic property.

### 3.3. Deformable Design

Most research focuses on fixed structural designs such as helical and spherical. However, some work has been performed on deformable designs to diversify MMRs functions or meet specific production needs. Those deformations are often triggered by external stimuli such as pH, ion strength, light, heat, and electric or magnetic fields [76]. For example, based on pH-responsive materials, Xin et al. reported an MMR, as shown in Figure 3f, capable of environmentally adaptive shape transformation through one-step 4D laser printing of heterogeneous structures within a single pH-responsive hydrogel. These MMRs could perform complex functions, including intricate microparticle manipulation (grasping, transporting, and releasing) [65]. To simplify the manufacturing process, some studies have exploited the phenomenon of self-folding caused by internal stresses generated between layers of different densities during 3D printing. This approach allows for printing 2D structures, which then automatically curl into 3D microrobots due to internal stresses, accelerating the production speed of robots. Darmawan et al. designed self-folding robots that could transform from 2D flat structures to helical MMRs within minutes [66]. Similarly, Nguyen et al. developed MMRs that self-fold from 2D planar structures into cylindrical shapes due to internal stress [67] (Figure 3g,h).

Although various structural designs have been explored, there are still challenges that can be addressed through innovative structural design. Lateral drift, for instance, occurs during forward motion. Conical hollow structures of MMRs have proven effective in reducing lateral drift [2]. Furthermore, the selective or independent control of MMRs can also be achieved through structural design [77]. However, most current structural designs of MMRs, tailored to their specific applications, rely on a trial-and-error approach. This process can be significantly accelerated through the use of machine learning methods.

## 4. Cargo Loading Methods

Before being deployed into the human body, MMRs must first load cargo. For drug agents, which are mostly chemical molecules, cargo Loading can be performed using physical and chemical methods such as mixing, adsorption, or covalent bonding. In contrast, loading cells typically involves culturing the cells on the MMRs, a process that ensures the cells adhere properly and maintain viability for delivery.

### 4.1. Physical Mixing

This process involves dispersing or embedding drugs within a hydrogel network, without establishing chemical bonds between the drug molecules and the carrier’s surface molecules. Usually, MNPs will be mixed with drugs to provide magnetism for microrobots.

Chen et al. mixed doxorubicin (DOX) (https://www.drugs.com/monograph/doxorubicin.html (accessed on 12 February 2024)) nanoparticles with MNPs and encapsulated them in hydrogel, subsequently creating orally administered child-parent MMRs using the extrusion dripping method [78]. The parent microrobot, whose material can not be hydrolyzed by a simulated gastric fluid, acted as a protective shell in the acidic environment of the stomach. This design prevented the release of DOX from the child microrobot in gastric fluid. When the robot reached the small intestine, the higher pH caused the parent microrobot to dissolve, leading to the drug release from the child microrobot. Similarly, Fusco et al. combined DOX and MNPs with a hydrogel matrix composed of N-isopropylacrylamide monomer (NIPAAM), acrylamide, polyethylene glycol diacrylate (PEGDA), and graphene oxide, which was solidified using photopolymerization to create a hydrogel-based drug delivery system with magnetic responsiveness and drug transport capabilities [52]. Ye et al. embeded DOX molecules, folate, and metal–organic frameworks (MOFs). MOFs served as the magnetic core within MMRs and were incorporated into a gelatin methacrylate (GelMA) hydrogel network, which served as the photoresist during the 3D printing process of these microrobots. The inclusion of folate enabled the MOFs to specifically recognize and bind to folate receptors on the surface of cancer cells, facilitating targeted therapy [79].

### 4.2. Physical Adsorption

In terms of the physical adsorption drug loading method, those drug molecules are directly adsorbed onto the surface of MMRs through weak Van der Waals forces or electrostatic interactions.

For example, utilizing the high affinity of DOX molecules for bismuth, Beladi-Mousavi et al. achieved rapid adsorption of DOX onto the MMRs’ surface by mixing the DOX solution with bismuth-based tubular MMRs [80]. Chitosan-based micro and nanoparticles are designed for targeted drug delivery as chitosan can absorb drug molecules [81]. Based on this, Chen et al. dispersed dried drug-free chitosan microspheres in a solution containing DOX or curcumin (CUR), allowing the drug to be loaded onto the chitosan microspheres through physical adsorption. The unadsorbed drug was removed by centrifugation and water washing, followed by drying to obtain the final drug-loaded chitosan microspheres, which were then encapsulated into robots [82]. Gong et al. used Chlamydomonas reinhardtii (Ch.) algal cells as templates of MMRs due to their micron-scale size, uniform spherical structure, and good biocompatibility. First, the cells (MMRs) were permeabilized and magnetized through chemical precipitation. The prepared cells were added to a phosphate-buffered saline solution containing DOX-HCl, allowing the DOX molecules to be physically adsorbed onto the surface of the cells [42]. In Darmawan et al.’s study, the DOX was combined with the surface of dopamine-modified MMRs. This combination relied on hydrogen bond interactions between the DOX molecules and the dopamine membrane, instead of forming stable chemical covalent bonds. The formation of non-covalent hydrogen bonds offered the possibility of the reversible loading of DOX, allowing for the controlled release of the drug under certain conditions, such as specific shear stress or pH changes [66]. Lee et al. used a temperature-responsive hydrogel, NIPAAM, as the main material for MMRs. By raising the temperature, MMRs were dehydrated (specifically the NIPAM component). This caused them to expand and increase in volume. Therefore, additional space was created for absorbing drugs. After immersing them in DOX, a subsequent decrease in temperature caused the MMRs to shrink, thereby enabling them to intake DOX [83].

### 4.3. Covalent Bonding

DOX contains multiple functional groups, such as quinone rings, hydroxyl groups, and amino groups, in its molecular structure, which enable DOX to form covalent bonds with a variety of chemical substances [84,85]. Thus, DOX can be conjugated with various MMRs through covalent bonding.

Villa et al. introduced semi-coated superparamagnetic polymer/iron oxide Janus particles, forming covalent bonds between the tosyl groups on the MMRs’ surface and the tetracycline structure of DOX’s amines. Additionally, the platinum (Pt) layer on the microrobots could further facilitate DOX binding through coordination with the hydroquinone part of the DOX molecule [86]. For biocompatibility considerations, Lee et al. mixed PEGDA with ethylenediamine to add amino groups to PEGDA, enabling PEGDA to form covalent bonds with DOX. After adding a photoinitiator, the solution was solidified under light exposure, and then DOX and MNPs were added to create a drug-loaded magnetic photoresist for printing microrobots through two-photon polymerization [48]. Malilick et al. activated the carboxyl groups (-COOH) on the surface of MMRs by using ethyl(dimethylaminopropyl) carbodiimide (EDC) and N-hydroxysuccinimide (NHS). The carboxyl groups formed stable covalent bonds with the amines (-NH_2_) in DOX, thereby fixing DOX to the MMRs [87]. Similarly, Song et al. introduced a magnetic tri-bead microrobot by mixing dicarboxylic azide compounds and biotin with NH_2_-Fe_3_O_4_ microbeads to prepare azide/biotin beads. By mixing azide/biotin beads with streptavidin-coated beads, a tri-bead/azide microrobot was created through the formation of stable covalent bonds between biotin and streptavidin. The N-H groups on DOX could bind with the free -COOH groups on the azide junctions, achieving covalent drug loading [88]. Genetically engineered strains of Escherichia coli MG1655, expressing biotin attachment peptides and green fluorescent protein, enabled the bacteria to covalently bind with MNPs through streptavidin. The biotin-streptavidin-biotin binding complex was further functionalized to integrate nanoliposome drug carrier units (i.e., liposomes) onto the live bacteria [89].

### 4.4. Cell Loading

Cell loading onto microrobots has become an innovative approach for various biomedical applications, including targeted therapy and regenerative medicine. Researchers have developed diverse methods for culturing and attaching cells to microrobots, each tailored to specific cell types and the intended applications. For instance, Gyak et al. developed a silicon carbonitride ceramic MMR. The successful culture of mouse fibroblast cells (NIH3T3) on MMRs demonstrates the excellent biocompatibility of silicon carbonitride ceramic. NIH3T3 was maintained in Dulbecco’s Modified Eagle Medium supplemented with 10% fetal bovine serum and 1% penicillin/streptomycin, and cultured at 37 °C in a 5% CO_2_ atmosphere. And finally, NIH3T3 was loaded to proposed MMRs [90]. In another study, fibroblast cells and mesenchymal stem cells (MSC) were maintained under similar conditions and then seeded on microrobots coated with poly-L-lysine (PLL). PLL was used because it was a positively charged synthetic amino acid chain that enhanced cell adhesion due to the negative charge of the cell membrane [74]. Noh et al. proposed a biodegradable spherical GelMA MMRs. Human nasal turbinate stem cells, cultured in α-MEM supplemented with 10% fetal bovine serum and 1% penicillin/streptomycin, were used as loaded cells for the testing of proposed MMRs [91]. Furthermore, hippocampal neural stem cells (NSCs) were cultured on surfaces coated with poly-l-ornithine and laminin. These NSCs were proved to be able to differentiate into neurons, oligodendrocytes, and astrocytes on different MMRs [92].

## 5. Cargo Release Methods

When MMRs reach the target area, they need to determine the most effective method to unload their cargo. For MMRs loaded with cells, arriving at the target area along with MMRs is sufficient. However, for drug-loaded MMRs, especially those carrying anticancer medications, precision in delivery is crucial. They must deliver their cargo to specific locations at designated times. This targeted delivery is designed to minimize the side effects of the drugs on humans.

### 5.1. Slow-Release

The most common method for drug release is the natural slow-release mechanism, which involves the dissolution of drug-encapsulating materials at certain pH levels to facilitate gradual release [93]. For example, Chen et al. designed a dual-layer drug-loaded MMR consisting of an outer layer of calcium alginate hydrogel and an inner layer of magnetic chitosan microspheres (mCS). Under a vision-guided magnetic propulsion system, the MMRs precisely delivered the drug-loaded mCS to the intended destination. The outer layer of MMRs protected the mCSs in acidic environments. In alkaline environments, the calcium alginate hydrogel dissolved at a controlled rate. This allowed for the sustained release of mCSs. As a result, the drug could be released not in the stomach’s acidic environment but in the intestine [94].

### 5.2. Activate Release

However, the slow-release methods lack control over drug release, which may result in premature or insufficient drug delivery [87]. Moreover, slow release is very time-consuming, and the MMRs may significantly change their position due to continuous propulsion, hindering the precision of targeted delivery [95].

**Figure 4:**
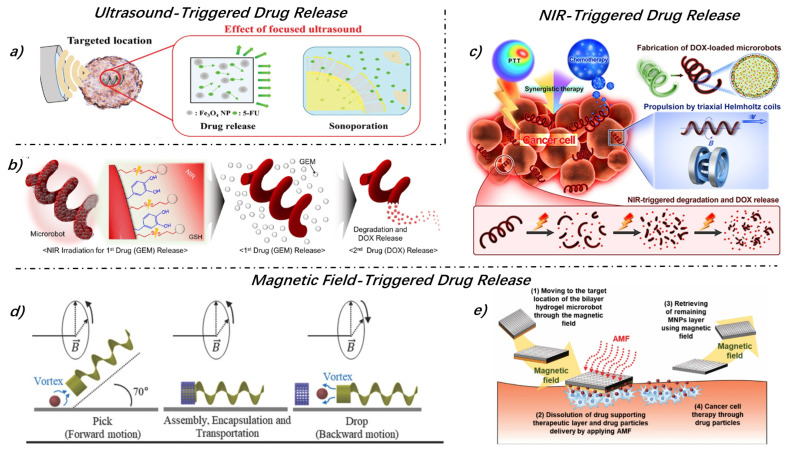
Active drug release by external stimuli. (**a**) Stable cavitation induced by ultrasound facilitates the release of drugs, leveraging acoustic energy to enhance delivery efficiency. (**b**) The first drug GEM is released through near-infrared (NIR) induced disulfide bond cleavage, then the second drug DOX is released by MMR degradation, enabling controlled sequential delivery. (**c**) NIR promotes the hydrolysis of MMRs, thereby triggering drug release. (**d**) Changing magnetic fields unscrew the embolus of MMRs to facilitate targeted release. (**e**) The application of alternating magnetic fields (AMFs) heats the MNPs layer of bilayer robots, subsequently melting the temperature-sensitive, drug-loaded gel layer to release the drug. Figures adapted with permissions from ref. [60], Wiley, (**a**); ref. [56], ACS, (**b**); ref. [101], ACS, (**c**); ref. [39], Wiley, (**d**); ref. [49], Wiley, (**e**).

Thus, many studies have favored using external stimuli for active drug release, with acoustic, light (mainly near-infrared, shorted as NIR), and magnetic field stimuli being widely used. For instance, as shown in Figure 4a, once the MMR reaches the target site, an ultrasonic beam would focus on the area to control the release of the payload [60]. Similarly, the long-short tubular MMR developed by Jeong et al. released drugs carried in the long tube by acoustic bubble excitation, removing the external bubble that covered the top of the tube as it approached the target tissue [46]. The ultrasonic treatment propagated to the hydrogel through compression and rarefaction cycles, causing gel failure and pore expansion in the hydrogel when the rarefaction exceeded the intermolecular attraction, leading to cell release [96]. Macrophages encapsulated in PVA@Fe3O4 hydrogels have also been shown to be actively released through therapeutic ultrasound triggering [97]. Except those mentioned, more examples of acoustic triggered drug release are well reviewed in [98]. While numerous studies demonstrate the efficacy of ultrasound methods, it is important to acknowledge that ultrasound may also have the potential to cause cellular damage [99].

NIR can generate heat, suitable for in vivo applications due to its ability to penetrate up to 10 cm of tissue with minimal absorption by skin and blood vessels [100]. When applied to thermo-sensitive hydrogel MMRs, NIR causes the robots to contract and dehydrate, further releasing the drug [83]. Wang et al. embedded Pd@Au core-shell nanoparticles into Spirulina, endowing the biological template with photothermal conversion capability, thus enabling drug release under NIR irradiation (Figure 4) [101]. Azo molecules, being thermo-sensitive, allow for the attachment of DOX to the surface of MNPs via azo linkages. Local heating by iron oxide nanoparticles under NIR irradiation triggers the decomposition of azo molecules and the release of DOX [102]. Lee et al. designed an MMR, as shown in Figure 4b, which was capable of sequentially releasing two drugs to enhance the efficacy of cancer cell treatment, selecting gemcitabine (GEM) and DOX as the first and second anticancer drugs to be released, respectively. GEM was linked to the surface of the robot via a disulfide bond, allowing it to be released first upon exposure to heat generated by NIR. Encapsulated within the robot by the physical mixing method, DOX was then gradually released through hydrogel degradation, offering a controlled delivery mechanism aimed at maximizing the therapeutic outcomes while the minimizing side effects [56].

Additionally, deformable MMRs proposed by Xin et al. can physically open and close in response to magnetic field changes, thereby releasing drugs [65]. Similar to this, the cap and plunger designed by Lee et al., as shown in Figure 4d, could unscrew the cap through changes in the magnetic field, thus releasing drugs [39]. Beyond the physical opening actions, temperature increases induced by eddy current losses under an alternating magnetic field could also trigger drug release. MMRs combining polypyrrole with low thermal conductivity and macromolecular dopants like polyethylene glycol have been shown to use alternating magnetic fields to accelerate drug release within the composite material [103]. Kim et al. developed a bilayer hydrogel composed of MNPs and therapeutic layers (Figure 4e), initially transported to tumor sites by an externally generated magnetic field. The application of an alternating magnetic field (AMF) increased the temperature of the bilayer hydrogel MMRs, causing the hydrogel to melt and release the drug [49].

Furthermore, electrochemical release mechanisms have also been explored [80]. Although injecting electrons into multilayer Bi/Ni/Pt microrobots enabled ultra-fast DOX release under neutral pH conditions within a couples of minutes, this experiment was only conducted in microfluidic channels with built-in electrochemical devices. So the feasibility of electro-reduction in the human body was very doubtful.

To provide a comprehensive comparison of the drug loading and release methodologies in recent MMRs research, Table 1 is presented. This table highlights a significant challenge in the field of MMRs for drug delivery: the lack of standardized metrics for evaluating drug loading and release rates. This issue is compounded by the fact that various studies utilize different experimental platforms, making direct comparisons almost impossible.

## 6. Tracking

For microrobotic cargo delivery, tracking techniques play a crucial role. The quick and precise tracking of microrobots ensures the real-time monitoring of microrobots’ movement, accurate navigation to disease sites. It also guarantees safety by facilitating post-treatment retrieval or degradation. Additionally, it aids in collecting and analyzing data to refine designs and improve treatment outcomes.

Whilst this article primarily focuses on magnetically actuated microrobots, it is worth mentioning tracking techniques, which have been tested on multiple microrobots platforms but can be compatible with magnetic actuation. In such a way, the scope of our article is expanded, and the described methods are adapted not only to the microrobotic systems designed for magnetic control but also for those microrobots that have other types of actuation.

Considering the micro size of MMRs, the integration of on-board tracking devices is often unrealistic due to the limited space and the impact on the microrobot’s functionality and maneuverability. For example, the dimensions of state-of-the-art on-board magneto-mechanical resonators used for miniature tracking are 1.9 mm in length and 0.8 mm in diameter, shaped like a small cylinder [105]. Although quite compact, it is still substantially larger than microrobots, which typically measure around 100 µm [74].

As a result, non-invasive tracking methods are utilized instead, such as magnetic resonance imaging (MRI), ultrasound, optical imaging, and nanoparticle labeling, catering to different treatment requirements. These techniques allow for precise localization and control of microrobots within the body without the need for embedding bulky or complex hardware.

### 6.1. Optical Tracking

Optical tracking often uses an endoscopic camera system for internal inspections, it offers a minimally invasive or non-invasive method to track microrobots. This approach provides high-resolution imaging, presenting a more cost-effective solution compared to other advanced imaging techniques such as MRI or computed tomography (CT) scans [106]. Zhang et al. used a monocular camera to capture the motion of microrobots with AI enhancement. Focus measurement techniques were used, which analyzed the camera’s focus information to infer depth. Gaussian depth residual neural networks were also implemented, which was a machine learning model designed to predict depth and posture information from single images. This approach was particularly useful in MMR cargo delivery where space or hardware limitations prevent the use of more complex multi-camera systems for depth and pose estimation [107]. However, using an optical endoscopic camera system to track microrobots presents several challenges. First, the field of view is generally limited, especially in complex environments where the microrobots might move out of the camera’s viewable area [108]. Second, lighting and visibility can also be problems, as in the complex environment poor lighting can lead to reduced accuracy. Reflections, shadows, and transparency within the working environment would further damage the visibility of microrobots, affecting the system’s overall efficacy [109]. As a result, the optical camera system is often used as a low-cost solution for testing microrobots in experimental environments such as microfluidic channels [110].

Fluorescent imaging methods use fluorescent materials in the fabrication of robots or the application of fluorescent markers post-production. It offers distinct advantages like low costs and simplicity in its application and is especially good at real-time tracking of microrobots. Lv et al. proposed a novel 3D tracking method for microrobots utilizing a fluorescent light field microscope, offering enhanced depth of field and 3D imaging from a single-shot image. This method significantly expanded the tracking capabilities, enabling the accurate determination of microrobots’ trajectories with high lateral resolution [111]. However, this approach was limited to the presence of auto-fluorescence in internal organs and bodily fluids, which interfered with the fluorescence from microrobots and led to errors. Thus, the fluorescent imaging method is often limited to superficial areas like the skin or surface of internal organs. Therefore, the imaging depth of normal fluorescence imaging is often limited up to 10 mm [112].

However, there are exceptions, such as the team led by Guanyin, who proposed the use of nanoparticles excited by near-infrared light, significantly increasing the contrast between targeted objects and background noise with a penetration depth of up to 3.2 cm [113]. The Xiangnan team proposed the DOLPHIN platform, which used NIR-II hyperspectral imaging and hyperdiffuse imaging modes in a trans-illumination configuration, allowing for the detection of fluorescent signals in deep tissues with a penetration depth of up to 8 cm [114]. Nevertheless, these high-penetration optical imaging methods had high costs and reduced flexibility due to the need for very specialized laser transmitters. Moreover, some fluorescent materials can be harmful to the human body and have difficulty integrating into the microrobots, such as quantum dots, making fluorescent imaging more challenging for clinical usage [115,116].

Optical coherence tomography (OCT) utilizes low-coherence light for high-resolution microrobot tracking. Light scattering from tissue and reflection from a reference mirror form an interference pattern, captured by a sensor. Adjusting the mirror enables depth scanning, producing detailed microrobot’s 3D position and pose [6]. Li’s study investigated OCT for in vivo microrobot navigation feedback, highlighting OCT’s advantages, including sub-100 μm imaging, micron-level resolution, and non-invasiveness for safe, real-time visualization. However, its key limitation is the limited penetration depth of only up to 2 mm, restricting deep tissue imaging. This is because as the depth of light penetration increases, tissue scattering and absorption also increase, therefore reducing the efficiency of light penetration and further leading to noise and a much lower resolution [6,117,118].

In general, optical tracking in microrobotics, using techniques like endoscopic cameras, fluorescent imaging, and OCT, provides precise and high-resolution tracking in real time. These systems are cost-effective but face challenges such as a limited field of view, lighting issues, and reduced visibility due to environmental reflections and shadows. These challenges are tied to the physical and optical properties of the environments and the technology itself, so they are difficult to fully address using only optical methods. Thus, integrating optical tracking systems with other technologies such as ultrasound and magnetic assist tracking is crucial for overcoming these limitations [119].

### 6.2. Ultrasonic Imaging

Ultrasonic imaging is a medical imaging technology that involves transmitting and receiving ultrasonic pulses from the tissues of the human body to create images. It benefits from the Doppler effect for accurate, real-time tracking of moving objects [120]. S. Pane et al. introduced magneto-responsive (MR) displacements as distinct markers for identifying objects in chaotic and dynamic environments. This method was tested by inducing MR vibrations using alternating magnetic fields. The vibrations were captured through ultrasound phase analysis, enabling high-contrast MR visualization and the extraction of features like size and position over time [121]. While the method enables fast imaging, it necessitates that the object’s size exceeds the ultrasound’s detection threshold. As a result, a compromise between the imaging resolution and imaging depth is required due to the inverse relationship between ultrasound’s decay and resolution with frequency, posing a challenge for deep tissue tracking [120]. The imaging depth of ultrasonic imaging varies depending on the frequency of the ultrasound waves: high-frequency ultrasonic waves (10–15 MHz) are suitable for superficial tissue levels, such as musculoskeletal and thyroid imaging, while low-frequency ultrasonic waves (2–5 MHz) are used for deeper tissue imaging, including deep abdominal, obstetric, and gynecological imaging [120], resulting in an acceptable potential imaging depth of 100 mm [116].

### 6.3. Magnetic Assist Tracking

MRI excels in visualizing MMRs, surpassing other imaging methods like CT, especially for in vitro and in vivo applications. Tiryaky et al. introduced a novel approach for 3D MMR tracking by utilizing deep learning techniques on 2D MRI captured during the microrobots’ motion. This method integrated a convolutional neural network with a complementary particle filter to achieve precise 3D tracking of microrobots. The key advantages of MRI include less damage to the human body, direct 3D section imaging without reconstruction, and superior imaging depth (more than 10 cm) contrast and resolution. However, its drawbacks are its high costs and lengthy imaging times [6,122].

Another magnetic assist tracking technique is the magnetic field camera. Vergne et al. described a system consisting of a 2D array of 3D magneto-resistive sensors. This setup enabled the tracking of the MMRs with a temporal resolution of 2 Hz. This system is notable for its simplicity, low cost, and relatively high precision. However, its penetrating depth is limited only for eye surgery, which is up to 30 mm. Also, it is not suitable for controlling the Z-axis of the robot and has limitations in the size of handled MMRs [123].

Despite significant advancements in magnetic assist tracking, such as the integration of deep learning with MRI for precise 3D tracking and the introduction of cost-effective, high-precision magnetic field cameras, there are some inherent shortages of magnetic assist tracking technique for tracking MMRs: it cannot track in real-time during manipulation due to magnetic field interference. This interference reduces imaging frequency to merely one or two frames per second, introducing substantial control risks and uncertainties, particularly in applications like cargo delivery.

### 6.4. Radioisotope Imaging

In the X-ray imaging field, metal particles that are integrated into microrobots as a contrast enhancer improve the contrast of robotic agents. This enhancement is important for enabling the precise motion tracking and imaging of microrobots. Nguyen et al. used a principal component analysis algorithm coupled with X-ray reconstruction for monitoring a microrobot’s position and orientation in real-time [124]. In medical applications, X-ray imaging can penetrate through the entire human body, providing effective imaging of bones and tissues [125].

Positron emission tomography (PET) and single-photon emission computed tomography (SPECT) are both nuclear medicine imaging tools. These methods combine microrobots with radioactive markers to release metal particles into X-ray imaging. Radioisotope imaging is characterized by a high spatial resolution and strong penetration power, making it simple to identify and track microrobots in the body [116,125].

Both PET and SPECT are highly effective for deep tissue imaging across the entire human body, depending on the power of gamma rays and radiotracers. In contrast, X-ray imaging’s effectiveness would vary significantly with tissue type. It requires additional adjustments to enhance the contrast in soft tissues. Nevertheless, these radioisotope imaging methods are also characterized by lengthy imaging times and high equipment costs, posing potential long-term risks to human health [6,116,125].

To give a clear comparison among tracking techniques, their advantages and limitations are summarized in Table 2 shown below.

### 6.5. Tracking of Cargo Distribution and Diffusion

Tracking cell distribution can be of great importance in cancer therapy as it allows for the precise monitoring of immune cells targeting cancer cells, helping to assess treatment effectiveness in real time. Zhao’s team developed NIR light-activated phthalocyanine-loaded lipid nanoparticles for labeling macrophages, enabling the efficient tracking of their homing to tumors [126]. Heo et al. proposed programmed nanoparticles to deliver antigens, and track the in vivo migration of dendritic cells after injection into the body through NIR imaging [127]. This focus on cell tracking in the in vivo environment has set a foundational precedent for the integration of similar imaging techniques in the field of microrobotics, where accuracy and safety are equally critical.

In the realm of MMRs, using imaging techniques to track cargo is of great clinical importance for precise drug therapy as well. Particularly, drug tracking can act as feedback control for MMRs, improving the treatment effectiveness and minimizing damage to tissues [128]. The tracking of cargo distribution via microrobots shares the same principles as tracking the microrobots, as they both are achieved by adding contrast agents to the tracked object to increase the contrast with the environment [129].

For example, in fluorescent imaging, NIR fluorescence imaging agents have emerged as the predominant choice for visualization. This preference is attributed to the ability of NIR photons to penetrate tissue and skin deeply with minimal background interference [130]. In MRI, paramagnetic ions, such as gadolinium, manganese, and iron, are typically employed as contrast agents to impart magnetic functionality [129,131,132,133,134].

However, since the cargo is for in vivo delivery, and could be challenging to degrade or retrieve from the human body. A critical focus must be on the safety of the contrast agents used. These contrast agents must be biocompatible and safe for long-term in vivo use to ensure that they do not cause damage to the human body while allowing for the effective monitoring of the drug delivery process. Therefore, the considerations for these contrast agents mirror those for microrobot tracking itself, while emphasizing the importance of non-toxicity and compatibility.

## 7. Navigation Algorithm

In the case of MMRs cargo delivery, ‘Navigation’ is the ability of these MMRs to move accurately in the human body. The importance of navigation in cargo delivery to patients is that it ensures that drugs are sent correctly to the lesion sites quickly, reducing the normal tissue damage caused by drugs as much as possible.

Navigation strategies in MMRs cargo delivery can be categorized as follows.

### 7.1. Dynamics Model-Based Navigation

Dynamics model-based navigation leverages pre-established physical and mathematical models to predict and control the motion of microrobots within the human body. This approach is distinguished by its precision and interpretability, offering a theoretically grounded method for navigating complex biological environments. However, its effectiveness is often limited by the inherent rigidity of the pre-established models, which struggle to adapt to the complexities of the in vivo environment. This limitation becomes particularly evident in extreme or unforeseen conditions, where the predefined models cannot accurately predict or respond to the dynamic changes [135].

Parvareh’s study introduced a navigation system accounting for a multitude of forces including drag, apparent gravity, electrostatic and contact forces, as well as magnetism. The control mechanism employed in this system utilized both backstepping and adaptive backstepping methods. These methods incrementally adjusted control inputs, thereby effectively minimizing system errors. This approach is particularly advantageous in scenarios characterized by parameter uncertainties, demonstrating its versatility in complex MMRs’ navigation [136]. Yang proposed an innovative double-loop control framework designed for the autonomous navigation of MMRs, particularly suited for complex fluidic environments. The inner loop focused on real-time tracking and positioning, ensuring the mechanism closely followed the MMRs. The outer loop utilized a combination of a disturbance observer, fuzzy logic modifier, and model predictive control to dynamically adjust magnetic fields for optimal steering to ensure precise manipulation and steering capabilities under varying conditions [137].

### 7.2. Machine Learning-Based Navigation

Navigation based on machine learning uses a data-driven approach to learn the optimal behavior of microrobots in the internal environment of the human body. The advantages of this method include: high robustness and adaptability [138]. However, the limitations of this approach mainly include that requires a large amount of high-quality data for training, especially for deep learning models, their decision-making process is often considered a ‘black box’, making it difficult to explain and verify [107].

In Salehi’s study, two model-free deep reinforcement learning control systems were developed to guide a disk-shaped MMRs in real-world scenarios. The systems employed the off-policy soft actor-critic algorithm and the on-policy trust region policy optimization algorithm for training. These approaches enabled the MMRs to autonomously learn and determine the most efficient routes to reach randomly assigned targets [32]. Abbasi et al.’s research illustrated the utilization of model-free reinforcement learning coupled with a gradual training strategy for controlling the three-dimensional positioning of the MMR within a predefined workspace. This approach involved directly manipulating coil currents. Significantly, their research introduced a novel training methodology that amalgamates simulation-based training with incrementally complex real-world scenarios. This progressive training method not only decreased the overall duration of training but also enhanced the accuracy of the MMR’s positioning [139]. Liu’s study delved into the challenges posed by the uncertainties and disturbances in MMRs’ navigation. The research addressed these challenges by dynamically updating the weights within a radial basis function neural network and adaptively modifying the gains in a sliding mode control system. This methodology ensured both the stability of the system and high navigation precision, circumventing the need for dynamic modeling of the system [138]. In the work of Behrens and Ruder, deep reinforcement learning was employed to develop robust control policies for helical MMRs autonomously. This research underscored the efficacy of reinforcement learning in automatically accounting for the real-world dynamics of the system. Such a model-free control approach substantially simplified the task for MMR engineers, enhancing the practicality of deploying these MMRs in various applications [140].

### 7.3. Multi-Level Adjustment Navigation

This category involves adjusting the navigation method based on the distances of the MMRs from the target site. The proposed hierarchical operational approach reduces the computational cost during the operation process and achieves highly accurate navigation control at crucial locations [141,142].

In the study by Lu et al., a novel multi-level magnetic delivery approach was introduced, combining a tethered microrobotic guidewire with untethered swimming microrobots. This integration effectively addressed the limitations inherent to each type, facilitating the robust and efficient delivery of microrobots in complex in vivo environments over substantial distances [71].

Conversely, Wang et al. proposed a control algorithm for these MMRs. This algorithm calculated both the distance and the alignment angle between the MMRs and its target, integrating these parameters as input variables for a drive force control mechanism. As the MMRs moved to the target, there was a proportional reduction in the driving force. The trajectory towards the target was segmented into discrete intervals, each peaking at designated target points. At these points, the MMR decelerated to a halt. This approach to control was optimized for speed and stability, aligning with the stringent requirements of the control process in cargo delivery robotics [142].

To give a clear comparison among the navigation techniques, some of their important parameters are concluded in the Table 3 below. It is noteworthy that some data were not directly provided in the text of those papers, so we had to estimate it from figures and other visual aids. Those data are noted by ^*^.

## 8. Degradation and Retrieval

In the post-cargo release phase within the human body, it is essential to consider how MMRs can be retrieved or degraded to mitigate potential side effects, such as infections. The integration of biodegradable materials into the construction of biocompatible MMRs has been a significant focus to address this concern. Currently, several biocompatible and biodegradable hydrogels have been employed in practical applications, including monodisperse calcium alginate hydrogel [78,143], poly(N-isopropylacrylamide) [52,83], gelatin methacrylate [79], poly(ethylene glycol) diacrylate (PEGDA) [48,144], and gelatin [145]. Copolymers such as PLGA [75] and degradable sugar-based materials like chitosan [82] and sucrose [59] have also been utilized.

**Figure 5:**
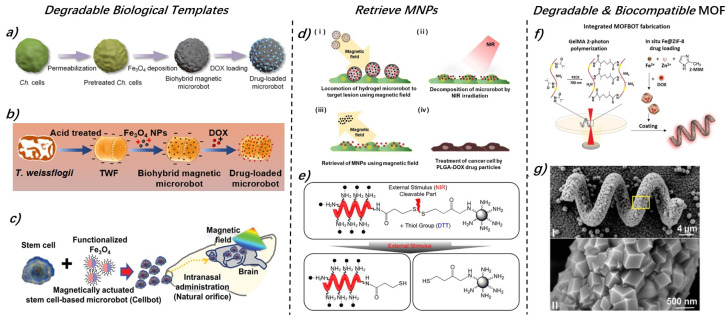
The design for degradation and retrieval of MMRs with biocompatibility consideration. (**a**–**c**) MMRs fabricated through biological templates and the adsorption and mixing method with MNPs, are, respectively, based on Chlamydomonas reinhardtii (Ch.), diatoms (TWF), and stem cells. These biological templates offer excellent biocompatibility and in vivo degradability. (**d**) NIR facilitates the degradation of MMRs, allowing for the retrieval of MNPs through a magnetic field. (**e**) NIR stimulation actively triggers the dissociation of MNPs from the robot’s surface through disulfide bond cleavage. Then MNPs can be retrieved by magnetic field. (**f**,**g**) Given the potential harm of MNPs to the human body, new MOF materials have been proposed. These materials boast superior biocompatibility and degradability in acidic solutions. Figures adapted with permissions from ref. [42], ACS, (**a**); ref. [104], Elsevier, (**b**); ref. [149], Wiley, (**c**); ref. [145], Elsevier, (**d**); ref. [48], ACS, (**e**); ref. [150], Wiley, (**f**); ref. [151], Wiley, (**g**).

Biological templates serve as a base for constructing microrobots, offering biocompatibility while simplifying the production process. After chemical modification, these biological templates can degrade within the body. Examples include Chlorella (Figure 5a) [42], Spirulina microalgae [101,146,147], pollen [148], diatoms (TWF) (Figure 5b) [104], Escherichia coli MG1655 [89], and stem cells (Figure 5c) [149].

Despite the excellent biocompatibility and degradability of these materials, nearly all these microrobots are constructed using Fe_3_O_4_ MNPs, integrating them into hydrogels, photoresists, or biological templates for further processing. This approach allows the microrobots to be magnetically driven. However, the considerable amount of MNPs in the microrobots may remain in the body post-therapy, potentially affecting the cellular metabolism or causing side effects such as membrane integrity disruption or apoptosis. Specifically, the reaction between iron ions and hydrogen peroxide in the biological environment can generate reactive oxygen species, reducing essential antioxidants and leading to normal cell death. Additionally, reactive oxygen species can produce toxic substances like aldehydes, damaging proteins [152]. MNPs may also cause intraocular hemorrhage and age-related macular degeneration, which are responsible for eye diseases such as retinal detachment and glaucoma [33].

To address this issue, some studies have explored retrieving MNPs post-drug release. Some approaches involved adding MNPs to the robots through physical mixing, allowing for magnetic field-based retrieval after drug release (Figure 5d) [145]. To actively control the timing of MNP retrieval, as shown in Figure 5e, Lee et al. linked MNPs to the robot surface via disulfide bonds, which break upon NIR irradiation, causing the MNPs to detach for magnetic retrieval [48]. Additionally, to tackle the issue of MNP biocompatibility, zeolitic imidazole framework-8 (ZIF-8, a type of MOF, Figure 5f,g) was proposed as a biocompatible material. Like MNPs, ZIF-8 possesses magnetic properties and can degrade in acidic environments, making it suitable for drug delivery applications in cancer treatment [151,153].

It is clear that the degradation and retrieval of MMRs for in vivo cargo delivery depend heavily on advancements in material science. The development of new biocompatible materials with magnetic properties or the ability to be magnetized would significantly enhance MMR technology. Therefore, interdisciplinary communication is essential for the advancement of MMRs. Additionally, utilizing machine learning to search for new biocompatible materials could further stimulate the development of MMRs for in vivo cargo delivery.

## 9. Challenges and Opportunities

Several unresolved challenges in the field are identified here after reviewing the developments in MMRs for in vivo cargo delivery since 2019. Accordingly, we provide potential solutions and offer insights to overcome these obstacles.

The actuation of MMRs typically faces challenges associated with their dexterous manipulation. The precise control of MMRs depends on the stability of the magnetic field, whether it is a static uniform field, a gradient field, or a rotating field generated by an alternating electric field. These fields are generally confined within a coil system or an electromagnetic array, which often has a considerably smaller working space compared to the volume of the actuation system itself. This limitation restricts the practical deployment of MMRs in medical scenarios, particularly for cargo delivery systems. Furthermore, the inherent global nature of magnetic fields poses significant challenges for achieving the independent control of individual MMRs. Therefore, future research into MMR actuation should focus on expanding the working space within the existing system volume and overcoming the challenges of global control, as these areas hold considerable promise for enhancing the functionality and application of MMRs.

Currently, the design of MMRs is primarily based on empirical and trial-and-error approaches, which become particularly problematic within the complex and sensitive environment of the human body. The human body is filled with fluids that exhibit low Reynolds numbers, complicating the provision of sufficient theoretical support and guidance for the robots. The relationship between design and performance is often high-dimensional, nonlinear, stochastic, and largely unknown, making the precise analytical modeling of these robots in real-world working environments exceedingly difficult. This uncertainty means that even minor design or environmental changes can lead to significant performance deviations. With the increasing functional demands on MMRs, such as performing biopsies and cell manipulation for more complex operations, merely meeting the basic propulsion requirements is insufficient to overcome these challenges. In this context, recent advancements in AI technologies, such as generative design and trial-and-error-based reinforcement learning, offer new possibilities to address these design and performance challenges [154,155]. These technologies can explore a wide range of design spaces to identify optimized structures and operational strategies, potentially achieving more efficient and precise MMR designs.

Moreover, there is a shortage of assessing and standardizing the cargo carrying capacity, release efficiency, as well as the navigation efficiency of MMRs. The lack of a unified evaluation framework makes it difficult to directly compare results between different studies. Although the targeted delivery of DOX has been a focus of research, existing designs may not be applicable when the drug is switched, highlighting a lack of attention to the carrying capacity for different anticancer drugs.

Besides given the inherent advantages and disadvantages of each tracking method for MMRs, the future development direction of MMR tracking technology necessitates the involvement of composite imaging techniques. This approach aims to achieve the comprehensive tracking of MMRs, encompassing all aspects of their operation and interaction with their environment. By integrating multiple imaging modalities, researchers can leverage the strengths of each technique while mitigating their weaknesses. This multidisciplinary strategy will likely lead to significant advancements in the precision, efficiency, and functionality of MMR tracking systems. Fortunately, the limitations of the tracking methods can be addressed through the application of AI. For instance, Zhang’s work demonstrates that analyzing a single plane can determine a robot’s 6D posture and location, surpassing traditional tracking methods [107]. Using AI can significantly compensate for methodological shortcomings, improving tracking accuracy and efficiency. However, the current research on tracking algorithms is primarily confined to computer vision, indicating that future advancements will necessitate the development of more diverse algorithms.

In the previous MMR navigation study, machine learning-based navigation’s primary drawbacks include the need for extensive, high-quality data, and its ‘black box’ decision-making process, which complicates explanation and verification [107]. The second category, dynamics model-based navigation, is noted for its precision and interpretability. It relies on fixed physical and mathematical models, limiting adaptability in dynamic environments [135,136]. To overcome those limitations, integrating the two approaches could be a promising strategy. If navigation is based on the precise real-world dynamic model, the amount of training data could be reduced. Also, the algorithm is more interpretable, making it easier for further optimization. Besides, with the actual dynamic model incorporated, parameters that can be measured in real-time, such as local viscosity, blood circulation velocity etc., can be easily input into the dynamic model for achieving high precision.

While methods using biocompatible hydrogels and the retrieval of MNPs have been proposed to enhance biocompatibility, the practical application of these schemes remains highly dependent on advancements in materials science. With the continuous discovery of new biocompatible materials, there is anticipation for more innovations and breakthroughs in this field.

Lastly, considerations for the magnetic driving platform currently revolve around electromagnetic actuation systems, which are mostly confined to laboratory-scale experiments, with insufficient consideration for actual medical environment applications. This involves not just the size and convenience of operation of the devices, but also their adaptability to complex in vivo environments and the requirements for control precision.

## 10. Conclusions

In this review, we delve into the developments in magnetically actuated microrobots for in vivo cargo delivery over the past five years. We analyze this research direction from an engineering perspective, deconstructing current work, including robot structural design, drug and cell carrying and release strategies, robot propulsion, tracking and navigation methods, and biocompatibility-focused robot retrieval and degradation approaches. Based on an analysis of these achievements, we highlight the current challenges and future opportunities for development.

Despite advancements in micro-scale fabrication techniques, research on MMRs for in vivo cargo delivery is still in its early stages. The structural design of MMRs often relies on trial and error, suggesting a promising potential for machine learning to accelerate design processes that better adapt to the complexities of in vivo circulation systems and enhance their functionality. Similarly, machine learning-driven approaches for tracking, navigation, and biocompatible materials discovery could shape the next generation of microrobotics research. In terms of cargo loading and release methods, there is a lack of standardization, making it urgent to establish benchmarks for the loading and release capacity of MMRs to facilitate comparisons across different design methods. As dexterous manipulation of MMRs becomes increasingly necessary, there is a growing need for developing actuation fields with minimal system volume to enable independent control and fit the need in real applications. Finally, interdisciplinary research will be essential, as clinical requirements in real-world applications may differ significantly from those in experimental environments.

Our aim of this review is to provide a practical, unified, and seamless research framework for newcomers, inspiring innovation and promoting the prosperity of the field. We hope this article serves as a beacon for researchers in their quest for innovation, guiding them through the vast ocean of research on magnetically actuated microrobots. In the future, we plan to more comprehensively summarize past achievements and focus on exploring how machine learning can empower this research area. Especially beyond tracking and navigation, we intend to delve into the potential of AI in enhancing microrobot design optimization, performance evaluation, drug release control, and biocompatibility analysis. By integrating advanced AI technologies, we anticipate solving current technical challenges and advancing the application of magnetically controlled microrobots in modern medicine, paving new pathways for more precise and efficient therapeutic methods.

## Figures and Tables

**Table 1:** Summary of drug loading and release methods.

Work(s)	Loading Method	Main Materials	Fabrication Method	Loading Rate ↑	Loading Time ↓	Release Rate ↓	Release Method
Ye et al. [79]	Physical Mixing	GelMA + e@ZIF-8 with ABF-MOF(FA)-DOX	Photopolymerization	-	2.5 h	96 h	Acidic environment release
Chen et al. [78]	Physical Mixing	Calcium alginate hydrogel	Extrusion droplet method	-	-	200 min	Intestinal slow release
Fusco et al. [52]	Physical Mixing	NIPAAM + Graphene Oxide	Photopolymerization	-	-	40%/4 h	NIR response
Kim et al. [49]	Physical Mixing	PEGDA + PLGA-DOX + MNPs	Photopolymerization	-	-	6 min	AMFs heats response
Darmawan et al. [66]	Physical Adsorption	E-dent 400 photoresist + MNPs	Photopolymerization	0.45 µg/MMR	Overnight	≥40%/10 s	HIFU acoustic drive
Lee et al. [83]	Physical Adsorption	NIPAM + MNPs	Photopolymerization	-	-	80.8%/6 h	NIR
Beladi-Mousavi et al. [80]	Physical Adsorption	Bi, Ni, Pt	Electrodeposition	145% of geometric surface area	725 min	Minutes in neutral pH	Electroreduction
Chen et al. [82]	Physical Adsorption	Chitosan particles + MNPs	Electrodeposition	65.2% for CUR, 41.6% for DOX	-	DOX 60.1%/24 h; CUR 36.2%/24 h	Slow release
Gong et al. [42]	Physical Adsorption	Ch. + MNPs	Biological template	95% with 80 µg/mL DOX	-	15%/180 min	Acidic slow release
li et al. [104]	Physical Adsorption	Diatom + MNPs	Biological template	29.1%	-	60%/8 h	Acidic slow release
Villa et al. [86]	Covalent Bonding	Platinum + MNPs	Pt sputtering	36.4% ± 4.3%	24 h	-	-
Lee et al. [48]	Covalent Bonding	PEGDA + MNPs	Two-photon polymerization printing	-	-	87%/40 min	-
Malilick et al. [87]	Covalent Bonding	EDC + NHS + Paramagnetic beads	-	10 µeq/g	-	-	Protease activity release
Song et al. [88]	Covalent Bonding	Azo compound + NH_2_-Fe_3_O_4_ beads	-	5.3 µg/mg	-	50%/10 h	NIR response
Akolpoglu et al. [89]	Covalent Bonding	*E. coli* MG1655 + MNPs	Biological template	86.1%	-	50%/5 h	NIR response

↑ Higher values indicate better performance. ↓ Lower values indicate better performance.

**Table 2:** Summary of tracking for microrobots.

Tracking Method	Imaging Depth	Limitations	Benefits	Reference
Endoscopic camera imaging	-	No penetration, Limited field of view	Low cost, High spatial resolution, High temporal resolution	[106,107,108,109,110,119]
Fluorescent imaging	<10 mm	Generally low penetration, Hard for integration, Potentially harmful	Low cost, High spatial resolution, High Temporal Resolution	[106,111,112,115,116]
OCT imaging	<2 mm	Low penetration	Extreme High spatial resolution, Safe, High temporal resolution	[6,117,118]
MRI	>1000 mm	High cost, Low temporal resolution, Sensitive to magnetic interference,	Strong penetration, High spatial resolution	[6,116,122]
Magnetic field camera	<30 mm	Low temporal resolution, Sensitive to magnetic interference.	Low cost, High spatial resolution	[123]
Ultrasonic Imaging	<100 mm	Limited spatial resolution, Limited robot’s size	Low cost, High temporal resolution, Safe	[116,120,121]
PET and SPECT Imaging	>1000 mm	Low temporal resolution, Potentially harmful, Low spatial resolution	Strong penetration	[6,116,125]
X-ray Imaging	>1000 mm	Low temporal resolution, Potentially harmful	Relative strong penetration	[124,125]

**Table 3:** Summary of microrobot’s navigation.

Category	Author(s)	Navigation Method	Relative Error	Robot’s Length	Speed
Multi-level Adjustment Navigation	Wang et al. [142].	Expert control algorithm	15.3–48.3% robot length	600 μm	5 mm/s
	Lu et al. [71].	Multi-level magnetic control	57–88% robot length	25–35 μm	194.7 ± 27.5 μm/s
Machine Learning Based Navigation	Liu et al. [138].	Adaptive neural network integrated with a sliding mode control	11.1–33.3% robot length	2–3 mm ^*^	above 20 μm/s ^*^
	Abbasi et al. [139].	Reinforcement learning coupled with a gradual training strategy	50% robot length	800 μm	above 1.5 mm/s
	Salehi et al. [32].	Model-free deep reinforcement learning	-	1.5 mm	11.9 mm/s ^*^
Dynamics Model Based Navigation	Yang et al. [137].	Double-loop motion controller	98% robot length	900 μm	6.2 mm/s
	Parvareh et al. [136].	Adaptive backstepping methods	10% robot length ^*^	500 μm	less than 40 mm/s

## Data Availability

There were no new data created.

## References

[1] Alapan Y., Bozuyuk U., Erkoc P., Karacakol A.C., Sitti M. (2020). Multifunctional surface microrollers for targeted cargo delivery in physiological blood flow. Sci. Robot..

[2] Xin C., Yang L., Li J., Hu Y., Qian D., Fan S., Hu K., Cai Z., Wu H., Wang D. (2019). Conical hollow microhelices with superior swimming capabilities for targeted cargo delivery. Adv. Mater..

[3] Choi J., Hwang J., Kim J.Y., Choi H. (2021). Recent progress in magnetically actuated microrobots for targeted delivery of therapeutic agents. Adv. Healthc. Mater..

[4] Shah Z.H., Wu B., Das S. (2022). Multistimuli-responsive microrobots: A comprehensive review. Front. Robot. AI.

[5] Yoo J., Tang S., Gao W. (2023). Micro-and nanorobots for biomedical applications in the brain. Nat. Rev. Bioeng..

[6] Liu X., Jing Y., Xu C., Wang X., Xie X., Zhu Y., Dai L., Wang H., Wang L., Yu S. (2023). Medical Imaging Technology for Micro/Nanorobots. Nanomaterials.

[7] Qi S., Wang X., Chang K., Shen W., Yu G., Du J. (2022). The bright future of nanotechnology in lymphatic system imaging and imaging-guided surgery. J. Nanobiotechnol..

[8] Zhang D., Ren Y., Barbot A., Seichepine F., Lo B., Ma Z.C., Yang G.Z. (2022). Fabrication and optical manipulation of micro-robots for biomedical applications. Matter.

[9] Zhang D., Barbot A., Lo B., Yang G.Z. (2020). Distributed force control for microrobot manipulation via planar multi-spot optical tweezer. Adv. Opt. Mater..

[10] Schrage M., Medany M., Ahmed D. (2023). Ultrasound microrobots with reinforcement learning. Adv. Mater. Technol..

[11] Bira N., Dhagat P., Davidson J.R. (2020). A review of magnetic elastomers and their role in soft robotics. Front. Robot. AI.

[12] He Y., Wang L., Zhong L., Liu Y., Rong W. Transporting microobjects using a magnetic microrobot at water surfaces. Proceedings of the 2018 15th International Conference on Control, Automation, Robotics and Vision (ICARCV).

[13] Chesnitskiy A.V., Gayduk A.E., Seleznev V.A., Prinz V.Y. (2022). Bio-Inspired Micro-and Nanorobotics Driven by Magnetic Field. Materials.

[14] Silva A.K.A., Silva E.L., Egito E.S.T., Carriço A.S. (2006). Safety concerns related to magnetic field exposure. Radiat. Environ. Biophys..

[15] Tukmachev D., Lunov O., Zablotskii V., Dejneka A., Babic M., Syková E., Kubinová Š. (2015). An effective strategy of magnetic stem cell delivery for spinal cord injury therapy. Nanoscale.

[16] Abbes M., Belharet K., Mekki H., Poisson G. Permanent magnets based actuator for microrobots navigation. Proceedings of the 2019 IEEE/RSJ International Conference on Intelligent Robots and Systems (IROS).

[17] Nadour H., Bozorg Grayeli A., Poisson G., Belharet K. (2023). CochleRob: Parallel-Serial Robot to Position a Magnetic Actuator around a Patient’s Head for Intracochlear Microrobot Navigation. Sensors.

[18] Kummer M.P., Abbott J.J., Kratochvil B.E., Borer R., Sengul A., Nelson B.J. (2010). OctoMag: An electromagnetic system for 5-DOF wireless micromanipulation. IEEE Trans. Robot..

[19] Yesin K.B., Vollmers K., Nelson B.J. (2006). Modeling and control of untethered biomicrorobots in a fluidic environment using electromagnetic fields. Int. J. Robot. Res..

[20] Choi H., Choi J., Jang G., Park J.o., Park S. (2009). Two-dimensional actuation of a microrobot with a stationary two-pair coil system. Smart Mater. Struct..

[21] Tehrani M.D., Kim M.O., Yoon J. (2014). A novel electromagnetic actuation system for magnetic nanoparticle guidance in blood vessels. IEEE Trans. Magn..

[22] Choi H., Cha K., Choi J., Jeong S., Jeon S., Jang G., Park J.O., Park S. (2010). EMA system with gradient and uniform saddle coils for 3D locomotion of microrobot. Sens. Actuators A Phys..

[23] Belharet K., Folio D., Ferreira A. (2011). Three-dimensional controlled motion of a microrobot using magnetic gradients. Adv. Robot..

[24] Yu J., Jin D., Chan K.F., Wang Q., Yuan K., Zhang L. (2019). Active generation and magnetic actuation of microrobotic swarms in bio-fluids. Nat. Commun..

[25] Kim S.H., Hashi S., Ishiyama K. (2010). Methodology of dynamic actuation for flexible magnetic actuator and biomimetic robotics application. IEEE Trans. Magn..

[26] Gwisai T., Mirkhani N., Christiansen M.G., Nguyen T.T., Ling V., Schuerle S. (2022). Magnetic torque–driven living microrobots for increased tumor infiltration. Sci. Robot..

[27] Alcântara C.C., Kim S., Lee S., Jang B., Thakolkaran P., Kim J.Y., Choi H., Nelson B.J., Pané S. (2019). 3D fabrication of fully iron magnetic microrobots. Small.

[28] Rikken R.S., Nolte R.J., Maan J.C., van Hest J.C., Wilson D.A., Christianen P.C. (2014). Manipulation of micro-and nanostructure motion with magnetic fields. Soft Matter.

[29] Abbott J.J., Ergeneman O., Kummer M.P., Hirt A.M., Nelson B.J. (2007). Modeling magnetic torque and force for controlled manipulation of soft-magnetic bodies. IEEE Trans. Robot..

[30] Abbott J.J., Peyer K.E., Lagomarsino M.C., Zhang L., Dong L., Kaliakatsos I.K., Nelson B.J. (2009). How should microrobots swim?. Int. J. Robot. Res..

[31] Li Z., Li C., Dong L., Zhao J. (2021). A review of microrobot’s system: Towards system integration for autonomous actuation in vivo. Micromachines.

[32] Salehi A., Hosseinpour S., Tabatabaei N., Soltani Firouz M., Yu T. (2024). Intelligent Navigation of a Magnetic Microrobot with Model-Free Deep Reinforcement Learning in a Real-World Environment. Micromachines.

[33] He X., Hahn P., Iacovelli J., Wong R., King C., Bhisitkul R., Massaro-Giordano M., Dunaief J.L. (2007). Iron homeostasis and toxicity in retinal degeneration. Prog. Retin. Eye Res..

[34] Zheng L., Chen L.G., Huang H.B., Li X.P., Zhang L.L. (2016). An overview of magnetic micro-robot systems for biomedical applications. Microsyst. Technol..

[35] Avaneesh R. (2022). Actuation, Control and Localization of Untethered Magnetic Robots. Master’s Thesis.

[36] Griffiths D.J. (2023). Introduction to Electrodynamics.

[37] Sakar M., Schürle S., Erni S., Ullrich F., Pokki J., Frutiger D., Ergeneman O., Kratochvil B., Nelson B. Non-contact, 3d magnetic biomanipulation for in vivo and in vitro applications. Proceedings of the 2012 International Symposium on Optomechatronic Technologies (ISOT 2012).

[38] Ullrich F., Schuerle S., Pieters R., Dishy A., Michels S., Nelson B.J. Automated capsulorhexis based on a hybrid magnetic-mechanical actuation system. Proceedings of the 2014 IEEE International Conference on Robotics and Automation (ICRA).

[39] Lee S., Kim S., Kim S., Kim J.Y., Moon C., Nelson B.J., Choi H. (2018). A Capsule-Type Microrobot with Pick-and-Drop Motion for Targeted Drug and Cell Delivery. Adv. Healthc. Mater..

[40] Lee S., Kim J.Y., Kim J., Hoshiar A.K., Park J., Lee S., Kim J., Pané S., Nelson B.J., Choi H. (2020). A needle-type microrobot for targeted drug delivery by affixing to a microtissue. Adv. Healthc. Mater..

[41] Jeong J., Jang D., Kim D., Lee D., Chung S.K. (2020). Acoustic bubble-based drug manipulation: Carrying, releasing and penetrating for targeted drug delivery using an electromagnetically actuated microrobot. Sens. Actuators A Phys..

[42] Gong D., Celi N., Zhang D., Cai J. (2022). Magnetic biohybrid microrobot multimers based on chlorella cells for enhanced targeted drug delivery. ACS Appl. Mater. Interfaces.

[43] Abdelaziz M., Habib M. (2022). Electromagnetic actuation for a micro/nano robot in a three-dimensional environment. Micromachines.

[44] Salmanipour S., Diller E. Eight-degrees-of-freedom remote actuation of small magnetic mechanisms. Proceedings of the 2018 IEEE International Conference on Robotics and Automation (ICRA).

[45] Wang Q., Yang L., Zhang L. (2021). Micromanipulation using reconfigurable self-assembled magnetic droplets with needle guidance. IEEE Trans. Autom. Sci. Eng..

[46] Jeong J., Jang D., Chung S.K. Target drug delivery technology (carrying, releasing, penetrating) using acoustic bubbles embedded in an electromagnetically driven microrobot. Proceedings of the 2018 IEEE Micro Electro Mechanical Systems (MEMS).

[47] Erni S., Schürle S., Fakhraee A., Kratochvil B.E., Nelson B.J. (2013). Comparison, optimization, and limitations of magnetic manipulation systems. J. Micro-Bio Robot..

[48] Lee H., Kim D.I., Kwon S.H., Park S. (2021). Magnetically actuated drug delivery helical microrobot with magnetic nanoparticle retrieval ability. ACS Appl. Mater. Interfaces.

[49] Kim D.I., Lee H., Kwon S.H., Sung Y.J., Song W.K., Park S. (2020). Bilayer hydrogel sheet-type intraocular microrobot for drug delivery and magnetic nanoparticles retrieval. Adv. Healthc. Mater..

[50] Shapiro B., Dormer K., Rutel I.B. (2010). A two-magnet system to push therapeutic nanoparticles. AIP Conf. Proc..

[51] Purcell E.M. (1977). Life at low Reynolds number. Am. J. Phys..

[52] Fusco S., Huang H.W., Peyer K.E., Peters C., Häberli M., Ulbers A., Spyrogianni A., Pellicer E., Sort J., Pratsinis S.E. (2015). Shape-switching microrobots for medical applications: The influence of shape in drug delivery and locomotion. ACS Appl. Mater. Interfaces.

[53] Peyer K.E., Zhang L., Nelson B.J. (2013). Bio-inspired magnetic swimming microrobots for biomedical applications. Nanoscale.

[54] Wang X., Hu C., Pané S., Nelson B.J. (2021). Dynamic modeling of magnetic helical microrobots. IEEE Robot. Autom. Lett..

[55] Samsami K., Mirbagheri S.A., Meshkati F., Fu H.C. (2020). Stability of soft magnetic helical microrobots. Fluids.

[56] Lee H., Park S. (2023). Magnetically Actuated Helical Microrobot with Magnetic Nanoparticle Retrieval and Sequential Dual-Drug Release Abilities. ACS Appl. Mater. Interfaces.

[57] Qiu F., Mhanna R., Zhang L., Ding Y., Fujita S., Nelson B.J. (2014). Artificial bacterial flagella functionalized with temperature-sensitive liposomes for controlled release. Sens. Actuators B Chem..

[58] Liu Y., Yang Y., Yang X., Yang L., Shen Y., Shang W. (2021). Multi-functionalized micro-helical capsule robots with superior loading and releasing capabilities. J. Mater. Chem. B.

[59] Gervasoni S., Terzopoulou A., Franco C., Veciana A., Pedrini N., Burri J.T., de Marco C., Siringil E.C., Chen X.Z., Nelson B.J. (2020). CANDYBOTS: A new generation of 3D-printed sugar-based transient small-scale robots. Adv. Mater..

[60] Park J., Kim J.y., Pane S., Nelson B.J., Choi H. (2021). Acoustically mediated controlled drug release and targeted therapy with degradable 3D porous magnetic microrobots. Adv. Healthc. Mater..

[61] Katsamba P., Lauga E. (2016). Micro-tug-of-war: A selective control mechanism for magnetic swimmers. Phys. Rev. Appl..

[62] Giltinan J., Katsamba P., Wang W., Lauga E., Sitti M. (2020). Selectively controlled magnetic microrobots with opposing helices. Appl. Phys. Lett..

[63] Wei T., Liu J., Li D., Chen S., Zhang Y., Li J., Fan L., Guan Z., Lo C.M., Wang L. (2020). Development of Magnet-Driven and Image-Guided Degradable Microrobots for the Precise Delivery of Engineered Stem Cells for Cancer Therapy. Small.

[64] Go G., Han J., Zhen J., Zheng S., Yoo A., Jeon M.J., Park J.O., Park S. (2017). A magnetically actuated microscaffold containing mesenchymal stem cells for articular cartilage repair. Adv. Healthc. Mater..

[65] Xin C., Jin D., Hu Y., Yang L., Li R., Wang L., Ren Z., Wang D., Ji S., Hu K. (2021). Environmentally adaptive shape-morphing microrobots for localized cancer cell treatment. ACS Nano.

[66] Darmawan B.A., Lee S.B., Go G., Nguyen K.T., Lee H.S., Nan M., Hong A., Kim C.S., Li H., Bang D. (2020). Self-folded microrobot for active drug delivery and rapid ultrasound-triggered drug release. Sens. Actuators B Chem..

[67] Nguyen K.T., Go G., Jin Z., Darmawan B.A., Yoo A., Kim S., Nan M., Lee S.B., Kang B., Kim C.S. (2021). A Magnetically Guided Self-Rolled Microrobot for Targeted Drug Delivery, Real-Time X-ray Imaging, and Microrobot Retrieval. Adv. Healthc. Mater..

[68] Folio D., Ferreira A. (2022). Modeling and Estimation of Self-Phoretic Magnetic Janus Microrobot With Uncontrollable Inputs. IEEE Trans. Control Syst. Technol..

[69] Arcese L., Fruchard M., Ferreira A. (2013). Adaptive controller and observer for a magnetic microrobot. IEEE Trans. Robot..

[70] Xie H., Sun M., Fan X., Lin Z., Chen W., Wang L., Dong L., He Q. (2019). Reconfigurable magnetic microrobot swarm: Multimode transformation, locomotion, and manipulation. Sci. Robot..

[71] Lu K., Zhou C., Li Z., Liu Y., Wang F., Xuan L., Wang X. (2024). Multi-level magnetic microrobot delivery strategy within a hierarchical vascularized organ-on-a-chip. Lab Chip.

[72] Rezende R.A., Pereira F.D., Kasyanov V., Ovsianikov A., Torgensen J., Gruber P., Stampfl J., Brakke K., Nogueira J.A., Mironov V. (2012). Design, physical prototyping and initial characterisation of ‘lockyballs’ This paper reports the fabrication of interlockable microscale scaffolds using two photon polymerization (2PP) and proposes a “lockyball” approach for tissue self-assembly for biofabrication. Virtual Phys. Prototyp..

[73] Danilevicius P., Rezende R.A., Pereira F.D., Selimis A., Kasyanov V., Noritomi P.Y., da Silva J.V., Chatzinikolaidou M., Farsari M., Mironov V. (2015). Burr-like, laser-made 3D microscaffolds for tissue spheroid encagement. Biointerphases.

[74] Li J., Li X., Luo T., Wang R., Liu C., Chen S., Li D., Yue J., Cheng S.H., Sun D. (2018). Development of a magnetic microrobot for carrying and delivering targeted cells. Sci. Robot..

[75] Go G., Jeong S.G., Yoo A., Han J., Kang B., Kim S., Nguyen K.T., Jin Z., Kim C.S., Seo Y.R. (2020). Human adipose–derived mesenchymal stem cell–based medical microrobot system for knee cartilage regeneration in vivo. Sci. Robot..

[76] Kirillova A., Ionov L. (2019). Shape-changing polymers for biomedical applications. J. Mater. Chem. B.

[77] Wang M., Wu T., Liu R., Zhang Z., Liu J. (2023). Selective and Independent Control of Microrobots in a Magnetic Field: A Review. Engineering.

[78] Chen W., Wen Y., Fan X., Sun M., Tian C., Yang M., Xie H. (2021). Magnetically actuated intelligent hydrogel-based child-parent microrobots for targeted drug delivery. J. Mater. Chem. B.

[79] Ye M., Zhou Y., Zhao H., Wang X. (2023). Magnetic Microrobots with Folate Targeting for Drug Delivery. Cyborg Bionic Syst..

[80] Beladi-Mousavi S.M., Khezri B., Krejcova L., Heger Z., Sofer Z., Fisher A.C., Pumera M. (2019). Recoverable bismuth-based microrobots: Capture, transport, and on-demand release of heavy metals and an anticancer drug in confined spaces. ACS Appl. Mater. Interfaces.

[81] Gao Y., Wu Y. (2022). Recent advances of chitosan-based nanoparticles for biomedical and biotechnological applications. Int. J. Biol. Macromol..

[82] Chen X., Zhang H., Tian C., Jia J., Xie H. (2022). Multiscale Magnetic Hydrogel Robot with a Core–Shell Structure for Active Targeted Delivery. ACS Appl. Polym. Mater..

[83] Lee H., Choi H., Lee M., Park S. (2018). Preliminary study on alginate/NIPAM hydrogel-based soft microrobot for controlled drug delivery using electromagnetic actuation and near-infrared stimulus. Biomed. Microdevices.

[84] Stuart C.H., Horita D.A., Thomas M.J., Salsbury F.R., Lively M.O., Gmeiner W.H. (2014). Site-specific DNA–Doxorubicin conjugates display enhanced cytotoxicity to breast cancer cells. Bioconjug. Chem..

[85] Oz Y., Barras A., Sanyal R., Boukherroub R., Szunerits S., Sanyal A. (2017). Functionalization of reduced graphene oxide via thiol–maleimide “click” chemistry: Facile fabrication of targeted drug delivery vehicles. ACS Appl. Mater. Interfaces.

[86] Villa K., Krejčová L., Novotnỳ F., Heger Z., Sofer Z., Pumera M. (2018). Cooperative multifunctional self-propelled paramagnetic microrobots with chemical handles for cell manipulation and drug delivery. Adv. Funct. Mater..

[87] Mallick S., Abouomar R., Rivas D., Sokolich M., Kirmizitas F.C., Dutta A., Das S. (2023). Doxorubicin-Loaded Microrobots for Targeted Drug Delivery and Anticancer Therapy. Adv. Healthc. Mater..

[88] Song X., Chen Z., Zhang X., Xiong J., Jiang T., Wang Z., Geng X., Cheang U.K. (2021). Magnetic tri-bead microrobot assisted near-infrared triggered combined photothermal and chemotherapy of cancer cells. Sci. Rep..

[89] Akolpoglu M.B., Alapan Y., Dogan N.O., Baltaci S.F., Yasa O., Aybar Tural G., Sitti M. (2022). Magnetically steerable bacterial microrobots moving in 3D biological matrices for stimuli-responsive cargo delivery. Sci. Adv..

[90] Gyak K.W., Jeon S., Ha L., Kim S., Kim J.y., Lee K.S., Choi H., Kim D.P. (2019). Magnetically Actuated SiCN-Based Ceramic Microrobot for Guided Cell Delivery. Adv. Healthc. Mater..

[91] Noh S., Jeon S., Kim E., Oh U., Park D., Park S.H., Kim S.W., Pané S., Nelson B.J., Kim J.Y. (2022). A biodegradable magnetic microrobot based on gelatin methacrylate for precise delivery of stem cells with mass production capability. Small.

[92] Jeon S., Kim S., Ha S., Lee S., Kim E., Kim S.Y., Park S.H., Jeon J.H., Kim S.W., Moon C. (2019). Magnetically actuated microrobots as a platform for stem cell transplantation. Sci. Robot..

[93] Li Y., Dong D., Qu Y., Li J., Chen S., Zhao H., Zhang Q., Jiao Y., Fan L., Sun D. (2023). A Multidrug Delivery Microrobot for the Synergistic Treatment of Cancer. Small.

[94] Chen W., Sun M., Fan X., Xie H. (2020). Magnetic/pH-sensitive double-layer microrobots for drug delivery and sustained release. Appl. Mater. Today.

[95] Heister E., Neves V., Tîlmaciu C., Lipert K., Beltrán V.S., Coley H.M., Silva S.R.P., McFadden J. (2009). Triple functionalisation of single-walled carbon nanotubes with doxorubicin, a monoclonal antibody, and a fluorescent marker for targeted cancer therapy. Carbon.

[96] Liu Y., Wang S., Lan W., Qin W. (2019). Development of ultrasound treated polyvinyl alcohol/tea polyphenol composite films and their physicochemical properties. Ultrason. Sonochem..

[97] Wu J., Liu L., Chen B., Ou J., Wang F., Gao J., Jiang J., Ye Y., Wang S., Tong F. (2021). Magnetically powered helical hydrogel motor for macrophage delivery. Appl. Mater. Today.

[98] Tu L., Liao Z., Luo Z., Wu Y.L., Herrmann A., Huo S. (2021). Ultrasound-controlled drug release and drug activation for cancer therapy. Exploration.

[99] Suslick K.S. (1988). Ultrasound: Its Chemical, Physical, and Biological Effects.

[100] Weissleder R. (2001). A clearer vision for in vivo imaging. Nat. Biotechnol..

[101] Wang X., Cai J., Sun L., Zhang S., Gong D., Li X., Yue S., Feng L., Zhang D. (2019). Facile fabrication of magnetic microrobots based on spirulina templates for targeted delivery and synergistic chemo-photothermal therapy. ACS Appl. Mater. Interfaces.

[102] Chen L., Wu L., Liu F., Qi X., Ge Y., Shen S. (2016). Azo-functionalized Fe_3_O_4_ nanoparticles: A near-infrared light triggered drug delivery system for combined therapy of cancer with low toxicity. J. Mater. Chem. B.

[103] Sivaraman K.M., Chatzipirpiridis G., Becsek B., Lühmann T., Ergeneman O., Nelson B.J., Pané S. Functional polypyrrole coatings for wirelessly controlled magnetic microrobots. Proceedings of the 2013 IEEE Point-of-Care Healthcare Technologies (PHT).

[104] Li M., Wu J., Lin D., Yang J., Jiao N., Wang Y., Liu L. (2022). A diatom-based biohybrid microrobot with a high drug-loading capacity and pH-sensitive drug release for target therapy. Acta Biomater..

[105] Gleich B., Schmale I., Nielsen T., Rahmer J. (2023). Miniature magneto-mechanical resonators for wireless tracking and sensing. Science.

[106] Wang B., Chan K.F., Yuan K., Wang Q., Xia X., Yang L., Ko H., Wang Y.X.J., Sung J.J.Y., Chiu P.W.Y. (2021). Endoscopy-assisted magnetic navigation of biohybrid soft microrobots with rapid endoluminal delivery and imaging. Sci. Robot..

[107] Zhang D., Lo F.P.W., Zheng J.Q., Bai W., Yang G.Z., Lo B. (2020). Data-driven microscopic pose and depth estimation for optical microrobot manipulation. ACS Photonics.

[108] Turan M., Pilavci Y.Y., Ganiyusufoglu I., Araujo H., Konukoglu E., Sitti M. (2018). Sparse-then-dense alignment-based 3D map reconstruction method for endoscopic capsule robots. Mach. Vis. Appl..

[109] Luo X., Xie L., Zeng H.Q., Wang X., Li S. (2023). Monocular endoscope 6-DoF tracking with constrained evolutionary stochastic filtering. Med. Image Anal..

[110] Hong A., Zeydan B., Charreyron S., Ergeneman O., Pané S., Toy M.F., Petruska A.J., Nelson B.J. (2017). Real-Time Holographic Tracking and Control of Microrobots. IEEE Robot. Autom. Lett..

[111] Lv J., Hu Y., Zhao H., Ye M., Ding N., Zhong J., Wang X. (2023). High-resolution and high-speed 3D tracking of microrobots using a fluorescent light field microscope. Quant. Imaging Med. Surg..

[112] Ding F., Feng J., Zhang X., Sun J., Fan C., Ge Z. (2021). Responsive optical probes for deep-tissue imaging: Photoacoustics and second near-infrared fluorescence. Adv. Drug Deliv. Rev..

[113] Chen G., Shen J., Ohulchanskyy T.Y., Patel N.J., Kutikov A., Li Z., Song J., Pandey R.K., Ågren H., Prasad P.N. (2012). (*α*-NaYbF_4_:Tm^3+^)/CaF_2_ Core/Shell Nanoparticles with Efficient Near-Infrared to Near-Infrared Upconversion for High-Contrast Deep Tissue Bioimaging. ACS Nano.

[114] Dang X., Bardhan N.M., Qi J., Gu L., Eze N.A., Lin C.W., Kataria S., Hammond P.T., Belcher A.M. (2019). Deep-tissue optical imaging of near cellular-sized features. Sci. Rep..

[115] Derfus A.M., Chan W.C., Bhatia S.N. (2004). Probing the cytotoxicity of semiconductor quantum dots. Nano Lett..

[116] Wang B., Zhang Y., Zhang L. (2018). Recent progress on micro-and nano-robots: Towards in vivo tracking and localization. Quant. Imaging Med. Surg..

[117] Li D., Dong D., Lam W., Xing L., Wei T., Sun D. (2019). Automated in vivo navigation of magnetic-driven microrobots using OCT imaging feedback. IEEE Trans. Biomed. Eng..

[118] Psomadakis C.E., Marghoob N., Bleicher B., Markowitz O. (2021). Optical coherence tomography. Clin. Dermatol..

[119] Zhang Y., Wang J., Yu H., Zheng J., Zhao X., Guo H., Qiu Y., Wang X., Liu L., Li W.J. (2023). A chemotactic microrobot with integrated iridescent surface for optical-tracking. Chem. Eng. J..

[120] Wang Q., Zhang L. (2020). Ultrasound imaging and tracking of micro/nanorobots: From individual to collectives. IEEE Open J. Nanotechnol..

[121] Pane S., Iacovacci V., Sinibaldi E., Menciassi A. (2021). Real-time imaging and tracking of microrobots in tissues using ultrasound phase analysis. Appl. Phys. Lett..

[122] Tiryaki M.E., Demir S.O., Sitti M. (2022). Deep learning-based 3D magnetic microrobot tracking using 2D MR images. IEEE Robot. Autom. Lett..

[123] Vergne C., Inácio J., Quirin T., Sargent D., Madec M., Pascal J. (2023). Tracking of a magnetically navigated millirobot with a magnetic field camera. IEEE Sens. J..

[124] Nguyen P.B., Kang B., Bappy D., Choi E., Park S., Ko S.Y., Park J.O., Kim C.S. (2018). Real-time microrobot posture recognition via biplane X-ray imaging system for external electromagnetic actuation. Int. J. Comput. Assist. Radiol. Surg..

[125] Rawson S.D., Maksimcuka J., Withers P.J., Cartmell S.H. (2020). X-ray computed tomography in life sciences. BMC Biol..

[126] Huang Y., Guan Z., Dai X., Shen Y., Wei Q., Ren L., Jiang J., Xiao Z., Jiang Y., Liu D. (2021). Engineered macrophages as near-infrared light activated drug vectors for chemo-photodynamic therapy of primary and bone metastatic breast cancer. Nat. Commun..

[127] Heo M.B., Lim Y.T. (2014). Programmed nanoparticles for combined immunomodulation, antigen presentation and tracking of immunotherapeutic cells. Biomaterials.

[128] Zhang Y., Yu J., Kahkoska A.R., Gu Z. (2017). Photoacoustic drug delivery. Sensors.

[129] Zheng F., Xiong W., Sun S., Zhang P., Zhu J.J. (2019). Recent advances in drug release monitoring. Nanophotonics.

[130] Kumar S., Richards-Kortum R. (2006). Optical molecular imaging agents for cancer diagnostics and therapeutics. Nanomedicine.

[131] Caravan P., Ellison J.J., McMurry T.J., Lauffer R.B. (1999). Gadolinium(III) Chelates as MRI Contrast Agents: Structure, Dynamics, and Applications. Chem. Rev..

[132] Klasson A., Ahrén M., Hellqvist E., Söderlind F., Rosén A., Käll P., Uvdal K., Engström M. (2008). Positive MRI contrast enhancement in THP-1 cells with Gd_2_O_3_ nanoparticles. Contrast Media Mol. Imaging.

[133] Zhang Y., Lin J.D., Vijayaragavan V., Bhakoo K.K., Tan T.T.Y. (2012). Tuning sub-10 nm single-phase NaMnF_3_ nanocrystals as ultrasensitive hosts for pure intense fluorescence and excellent T_1_ magnetic resonance imaging. Chem. Commun..

[134] Weissleder R., Elizondo G., Wittenberg J., Rabito C.A., Bengele H.H., Josephson L. (1990). Ultrasmall superparamagnetic iron oxide: Characterization of a new class of contrast agents for MR imaging. Radiology.

[135] Kararsiz G., Duygu Y.C., Wang Z., Rogowski L.W., Park S.J., Kim M.J. (2023). Navigation and Control of Motion Modes with Soft Microrobots for Drug Delivery at Low Reynolds Numbers. Micromachines.

[136] Parvareh A., Ibrahimi F., Nasseri M.A. (2023). Nonlinear swimming magnetically driven microrobot influenced by a pulsatile blood flow through adaptive backstepping control having weight estimation. Int. J. Dyn. Control.

[137] Yang Z., Yang L., Zhang L. (2021). Autonomous navigation of magnetic microrobots in a large workspace using mobile-coil system. IEEE/ASME Trans. Mechatron..

[138] Liu Y., Wang H., Fan Q. (2023). Adaptive learning and sliding mode control for a magnetic microrobot precision tracking with uncertainties. IEEE Robot. Autom. Lett..

[139] Abbasi S.A., Ahmed A., Noh S., Gharamaleki N.L., Kim S., Chowdhury A.M.B., Kim J.Y., Pané S., Nelson B.J., Choi H. (2024). Autonomous 3D positional control of a magnetic microrobot using reinforcement learning. Nat. Mach. Intell..

[140] Behrens M.R., Ruder W.C. (2022). Smart magnetic microrobots learn to swim with deep reinforcement learning. Adv. Intell. Syst..

[141] Zhu Y.X., Jia H.R., Jiang Y.W., Guo Y., Duan Q.Y., Xu K.F., Shan B.H., Liu X., Chen X., Wu F.G. (2023). A red blood cell-derived bionic microrobot capable of hierarchically adapting to five critical stages in systemic drug delivery. Exploration.

[142] Wang J., Jiao N., Tung S., Liu L. (2016). Automatic path tracking and target manipulation of a magnetic microrobot. Micromachines.

[143] Sitti M., Ceylan H., Hu W., Giltinan J., Turan M., Yim S., Diller E. (2015). Biomedical applications of untethered mobile milli/microrobots. Proc. IEEE.

[144] Park J., Jin C., Lee S., Kim J.Y., Choi H. (2019). Magnetically actuated degradable microrobots for actively controlled drug release and hyperthermia therapy. Adv. Healthc. Mater..

[145] Kim D.I., Lee H., Kwon S.H., Choi H., Park S. (2019). Magnetic nano-particles retrievable biodegradable hydrogel microrobot. Sens. Actuators B Chem..

[146] Yan X., Zhou Q., Vincent M., Deng Y., Yu J., Xu J., Xu T., Tang T., Bian L., Wang Y.X.J. (2017). Multifunctional biohybrid magnetite microrobots for imaging-guided therapy. Sci. Robot..

[147] Gong D., Cai J., Celi N., Feng L., Jiang Y., Zhang D. (2018). Bio-inspired magnetic helical microswimmers made of nickel-plated Spirulina with enhanced propulsion velocity. J. Magn. Magn. Mater..

[148] Sun M., Fan X., Meng X., Song J., Chen W., Sun L., Xie H. (2019). Magnetic biohybrid micromotors with high maneuverability for efficient drug loading and targeted drug delivery. Nanoscale.

[149] Jeon S., Park S.H., Kim E., Kim J.Y., Kim S.W., Choi H. (2021). A Magnetically Powered Stem Cell-Based Microrobot for Minimally Invasive Stem Cell Delivery via the Intranasal Pathway in a Mouse Brain. Adv. Healthc. Mater..

[150] Terzopoulou A., Wang X., Chen X.Z., Palacios-Corella M., Pujante C., Herrero-Martín J., Qin X.H., Sort J., deMello A.J., Nelson B.J. (2020). Biodegradable metal–organic framework-based microrobots (MOFBOTs). Adv. Healthc. Mater..

[151] Wang X., Chen X.Z., Alcântara C.C., Sevim S., Hoop M., Terzopoulou A., De Marco C., Hu C., de Mello A.J., Falcaro P. (2019). MOFBOTS: Metal–organic-framework-based biomedical microrobots. Adv. Mater..

[152] Shen Y., Huang Z., Liu X., Qian J., Xu J., Yang X., Sun A., Ge J. (2015). Iron-induced myocardial injury: An alarming side effect of superparamagnetic iron oxide nanoparticles. J. Cell. Mol. Med..

[153] Sun C.Y., Qin C., Wang X.L., Yang G.S., Shao K.Z., Lan Y.Q., Su Z.M., Huang P., Wang C.G., Wang E.B. (2012). Zeolitic imidazolate framework-8 as efficient pH-sensitive drug delivery vehicle. Dalton Trans..

[154] Jebellat I., Jebellat E., Amiri-Margavi A., Vahidi-Moghaddam A., Pishkenari H.N. (2024). A reinforcement learning approach to find optimal propulsion strategy for microrobots swimming at low reynolds number. Robot. Auton. Syst..

[155] Dhatt-Gauthier K., Livitz D., Wu Y., Bishop K.J. (2023). Accelerating the Design of Self-Guided Microrobots in Time-Varying Magnetic Fields. JACS Au.

